# Turnip mosaic virus selectively subverts a PR‐5 thaumatin‐like, plasmodesmal protein to promote viral infection

**DOI:** 10.1111/nph.20233

**Published:** 2024-11-12

**Authors:** Rongrong He, Yinzi Li, Mark A. Bernards, Aiming Wang

**Affiliations:** ^1^ London Research and Development Centre Agriculture and Agri‐Food Canada London ON N5V 4T3 Canada; ^2^ Department of Biology Western University 1151 Richmond St. London ON N6A 5B7 Canada

**Keywords:** *Arabidopsis thaliana*, *Nicotiana benthamiana*, osmotin, plasmodesmata (PD), potyvirus, reactive oxygen species (ROS), viral intercellular movement, virus replication complex (VRC)

## Abstract

- Pathogenesis‐related (PR) proteins are induced by abiotic and biotic stresses and generally considered as part of the plant defense mechanism. However, it remains yet largely unclear if and how they are involved in virus infection. Our recent quantitative, comparative proteomic study identified three PR‐5 family proteins that are significantly differentially accumulated in the plasmodesmata (PD)‐enriched fraction isolated from *Nicotiana benthamiana* leaves infected by turnip mosaic virus (TuMV).
- In this study, we employed the TuMV‐Arabidopsis pathosystem to characterize the involvement of two Arabidopsis orthologs, AtOSM34 and AtOLP of the three *N. benthamiana* PR‐5‐like proteins.
- We show that AtOSM34 and AtOLP are PD‐localized proteins and their expression is up‐ and downregulated in response to TuMV infection, respectively. Deficiency or overexpression of AtOLP does not affect viral RNA accumulation. Knockdown of *AtOSM34* inhibits TuMV infection, whereas its overexpression promotes viral infection. We further demonstrate that AtOSM34 functions as a proviral factor through diminishing PD callose deposition to promote viral intercellular movement, targeting the viral replication complex to enhance viral replication, and suppressing the ROS‐mediated antiviral response.
- Taken together, these data suggest that TuMV has evolved the ability to selectively upregulate and subvert AtOSM34, a PR‐5 family protein to assist its infection.

## Introduction

Plant viruses infect almost all crops, causing serious diseases world‐wide and threatening global food security. Plant viruses usually have small genomes that encode a few proteins and, thus, largely depend on host cellular machinery and factors to complete their infection cycle (Nagy & Pogany, 2012; Wang, 2015; Hyodo & Okuno, 2020; He *et al.*, 2023). Unlike animal viruses that enter adjacent cells through receptor‐mediated endocytosis or membrane fusion, plant viruses move to neighboring cells through plasmodesmata (PD) to establish systemic infection. PD are plasma membrane‐lined pores, a specialized intercellular organelle that enables cytoplasmic and endomembrane continuity between adjacent cells (Cheval & Faulkner, 2018; Petit *et al*., 2020; Li *et al*., 2021; Burch‐Smith, 2024; Tee & Faulkner, 2024). As intercellular channels, PD are dynamic structures, potentially allowing passage of small metabolites, assumably signal molecules and even macromolecules between neighboring cells, and the primary limiting factor of this capacity is the size of the plasmodesmal aperture (Nicolas *et al*., 2017; Tee & Faulkner, 2024). Due to this limitation, viral intercellular movement via PD requires the coordinated action of virus‐encoded proteins and host factors, especially PD‐localized ones (Wang, 2021). Molecular identification and functional characterization of PD‐specific host proteins may assist in the development of novel strategies to control plant viral diseases for the sustainable crop production (Liu *et al*., 2021).

Most known viruses have a positive‐sense single‐stranded (+ss) RNA genome. Potyviruses (viruses in the genus *Potyvirus* in the family *Potyviridae*) constitute the largest group of plant‐infecting +ssRNA viruses including several agriculturally and economically important ones such as potato virus Y, plum pox virus, soybean mosaic virus and turnip mosaic virus (TuMV) (Revers & García, 2015; Yang *et al*., 2021). The potyviral +ssRNA genome is an RNA of *c*. 10 kb that contains a single large open reading frame (ORF). During viral genome replication, transcriptional slippage at the coding region for the third protein (P3) leads to the generation of a small percentage of viral subpopulation whose genome encodes a short ORF due to frame shift (Cui & Wang, 2019; Yang *et al*., 2021). After translation, the long and short polyproteins are proteolytically processed into 11 mature proteins and various intermediate precursor proteins (Revers & García, 2015; Yang *et al*., 2021). Among them, the second 6‐kDa protein, 6K2 is an integral membrane protein that targets and remodels the endoplasmic reticulum (ER) to initiate the formation of ER‐derived membranous vesicles for viral replication (Schaad *et al*., 1997; Wei & Wang, 2008; Cotton *et al*., 2009).

To better understand the involvement of PD in viral infection, our lab conducted a quantitative, comparative proteomic study between the PD‐enriched fractions from leaf tissues of *Nicotiana benthamiana* L. plants infected by TuMV and healthy leaves of mock‐inoculated plants (Park *et al*., 2017). Three osmotin (OSM)‐like proteins (OLPs) were identified to be significantly differentially accumulated in TuMV‐infected leaves, compared to their amounts in the healthy control. Together with thaumatins, zeamatin and their like proteins (TLPs and ZLPs), OSMs and OLPs belong to the PR‐5 family (the fifth class of pathogenesis‐related proteins) (also known as TLP family) (Kumar *et al*., 2015; Hakim *et al*., 2018). The PR‐5 family proteins are produced in plants in response to various biotic and abiotic stresses and play important roles in the plant immune system to confer tolerance to them (Sinha *et al*., 2014). Osmotin has been recognized as a plant defense tool (reviewed by Hakim *et al*., 2018). In the present study, we used the Arabidopsis orthologs (AtOSM34 and AtOLP) of the three OLPs identified from *N*. *benthamiana* to investigate their possible roles in TuMV infection. We show that both AtOSM34 and AtOLP are PD‐located proteins and are differentially regulated in response to TuMV infection, but only AtOSM34 functions as a proviral factor. AtOSM34 is recruited to the viral replication complex (VRC) likely via its interaction with the TuMV 6K2 protein to promote viral replication. We further present evidence that AtOSM34 also promotes viral cell‐to‐cell movement. Moreover, we demonstrate that AtOMS34 suppresses antiviral resistance mediated by reactive oxygen species (ROS) burst.

## Materials and Methods

### Plant materials and growth conditions

The Arabidopsis (*Arabidopsis thaliana* (L.) Col.) mutants *atosm34* (CS916067), *atolp* (CS819509) and *atolp* (CS850431) used in this study were obtained from the Arabidopsis Biological Resource Centre (ABRC) at the Ohio State University (Columbus, USA), and screened for homozygous plants as described previously (Huang *et al*., 2010; Li *et al*., 2016b; Cheng *et al*., 2017) using primers listed in Supporting Information Table S1. Transgenic Arabidopsis (ecotype Col‐0) plants overexpressing AtOSM34 or AtOLP were generated by the floral‐dip method (Zhang *et al*., 2006). Transformants were screened by directly spraying of solutions containing 20 mg l^−1^ glufosinate‐ammonium, and further confirmed by PCR and immunoblotting. All *A. thaliana* and *N. benthamiana* plants were grown as described previously (Li *et al*., 2018; Wu *et al*., 2020).

### Gene cloning, plasmid construction and expression

The Phusion high‐fidelity DNA polymerase (New England Biolabs, Whitby, ON, Canada) was used for PCR to amplify all DNA sequences, and Gateway technology (Thermo Fisher Scientific, Mississauga, ON, Canada) was employed for plasmid construction. Coding sequences of *AtOSM34* from *A. thaliana* Col‐0 were amplified from complementary DNA (cDNA) derived from Arabidopsis leaf messenger RNAs (mRNAs). Coding sequences of TuMV genes were amplified from the infectious clones pCambiaTuMV::GFP (TuMV‐GFP) (Thivierge *et al*., 2008; Cotton *et al*., 2009). This recombinant TuMV infectious clone contains the coding sequence for green fluorescent protein (GFP) so that viral infection could be monitored by fluorescence. The cloned coding sequences were recombined into entry vector pDONR221 (Invitrogen) before final recombination to plant expression vectors by LR reactions. AtOSM34 were recombined into pEarleyGate101 and pEarleyGate104. For the bimolecular fluorescence complementation (BiFC) assay, cDNAs of interest were transferred into the modified 35S‐YN Gateway and 35S‐YC Gateway vectors (Lu *et al*., 2010) to construct plant transient expression plasmids expressing recombinant proteins tagged to the N‐terminal half of yellow fluorescent protein (YFP) (YN) or the C‐terminal half of YFP (YC). The pBA‐Flag‐4×Myc‐DC vector (Zhu *et al*., 2011) was used to generate Flag‐4×Myc‐tagged constructs. All constructs were verified by DNA sequencing to ensure that no errors were introduced during PCR amplification and plasmid construction. All plant expression vectors were electroporated into cells of *Agrobacterium tumefaciens* strain GV3101. The GV3101 cells harboring the expression constructs were resuspended in infiltration buffer (10 mM MgCl_2_, 10 mM MES (2‐morpholinoethanesulphonic acid), pH 5.6, and 100 μM acetosyringone) and agroinfiltrated into *N. benthamiana* leaves as described (Wei & Wang, 2008; Wei *et al*., 2010b).

### RNA extraction and RT‐qPCR

Total RNA was extracted from leaf tissues or protoplasts using the Plant Total RNA Mini Kit (Geneaid, New Taipei City, Taiwan) and reverse transcribed using the SuperScript III First‐Strand Synthesis System (Thermo Fisher Scientific) according to the manufacturer's instructions. Quantitative polymerase chain reaction was performed as described previously (Cheng *et al*., 2017; Park *et al*., 2017). Primers used in this study are listed in Table S1.

### Protoplast isolation and TuMV replication assay

Mesophyll protoplasts were prepared from 4‐wk‐old Arabidopsis leaves essentially as described (Deng *et al*., 2015; Dai & Wang, 2022a). About 1 × 10^5^ protoplasts were transfected with 20 μg TuMV‐GFP in polyethylene glycol (PEG)–calcium transfection solution (40% (w/v) PEG 4000, 0.2 M mannitol and 0.1 M CaCl_2_) at room temperature for 20 min. Transformed protoplasts were then washed and resuspended in W5 buffer (154 mm NaCl, 125 mm CaCl_2_, 5 mm KCl, 5 mM glucose and 2 mm MES, pH 5.7) and incubated at 25°C to allow virus replication. At 42 h posttransfection (hpt), protoplasts were harvested for RNA extraction. Transfection assays were repeated at least three times.

### Immunoblotting and co‐immunoprecipitation

Total proteins were extracted as described previously (Li *et al*., 2020), separated by SDS‐PAGE (Sodium dodecyl‐sulfate polyacrylamide gel electrophoresis), transferred to polyvinylidene difluoride membrane, and then blocked with 5% skimmed milk in PBST (137 mM NaCl, 2.7 mM KCl, 10 mM Na_2_HPO_4_, 1.8 mM KH_2_PO_4_ and 0.2% (v/v) Tween 20). Immunoblotting was performed essentially as described previously (Cheng *et al*., 2017; Li *et al*., 2020; Wu *et al*., 2020).

Co‐immunoprecipitation (co‐IP) experiments were carried out with anti‐Flag M2 gel as previously described (Win *et al*., 2011; G. Wu *et al*., 2018) with modifications. Briefly, viral proteins were overexpressed in *N. benthamiana* leaves using the modified high‐expression vector pSK104 with an N‐terminal 3 × Flag tag (Kagale *et al*., 2012). Host proteins of interest were overexpressed in *N. benthamiana* leaves using the pEarleyGate‐104 vector with an N‐terminal YFP tag (Earley *et al*., 2006). About 1 g of *N*. *benthamiana* leaves expressing different combinations of proteins were harvested for total protein extraction. The cleared lysates were then immunopurified using anti‐Flag M2 gel followed by immunoblot analysis using anti‐Flag monoclonal antibody and anti‐N‐terminal GFP polyclonal antibodies (Sigma‐Aldrich, Oakville, ON, Canada).

### Yeast‐two hybrid (Y2H) and BiFC assays

The split‐ubiquitin membrane‐based yeast‐two‐hybrid (mYTH) assay was performed using a DUAL membrane pairwise interaction kit (Dualsystems Biotech AG, Schlieren, Switzerland) according to the manufacturer's protocols. Briefly, *Saccharomyces cerevisiae* (strain NMY51) was co‐transformed with the recombinant pBT3‐STE and pPR3‐N plasmids and then grown on selection medium lacking leucine and tryptophan (SD/−Leu/−Trp) (Clontech, USA) at 28°C for up to 4 d. The growing colonies were then transferred to SD/−Leu/−Trp/−His/−Ade medium (Clontech, Mountain View, CA, USA) lacking leucine, tryptophan, histidine, and adenine supplemented with 10–50 mm 3‐AT (Sigma‐Aldrich).

For the BiFC assay, different combinations of vectors containing target genes for interaction identification were agroinfiltrated into *N. benthamiana* leaves as described previously (Li *et al*., 2018).

### Viral intercellular movement assay

TuMV cell‐to‐to movement was assayed in *N. benthamiana* leaves with TuMV‐GFP//mCherry‐HDEL essentially as described (Dai *et al*., 2020; Dai & Wang, 2022b). In brief, agrobacterial cells harboring TuMV CP mutants (OD_600_ 0.0001) were coinfiltrated with each of the OLP expression vectors (OD_600_ 0.3) into 3–4‐wk‐old *N. benthamiana* plants. Viral cell‐to‐cell movement was monitored by confocal microscopy from 2 to 4 d postinfiltration (dpi) at intervals of *c*. 8 h.

### Virus‐induced gene silencing (VIGS) assay

A 300‐bp fragment of *NbOSM* was amplified from *N. benthamiana* leaf cDNA using primers TRV‐NbOSM‐F and TRV‐NbOSM‐R (Table S1). The PCR product was inserted into the pTRV2 vector to generate pTRV2–NbOSM (Liu *et al*., 2002; Park *et al*., 2017). *Agrobacterium* cultures harboring pTRV1 and pTRV2–NbOSM were mixed in a ratio of 1 : 1 (v/v) and infiltrated into *N. benthamiana* leaves as described (Liu *et al*., 2002; Senthil‐Kumar & Mysore, 2014; Park *et al*., 2017). The accumulation level of *NbOSM* mRNA was determined by reverse transcription quantitative polymerase chain reaction at 10 dpi.

### Confocal microscopy

Confocal microscopy analysis of *N. benthamiana* leaves at 48–72 h postinoculation, unless otherwise specified, was carried out using an Olympus FV1200 laser scanning microscope (Olympus Corp., Tokyo, Japan) with excitation wavelength of 440 nm for CFP, 488 nm for GFP, 515 nm for YFP and 559 nm for mCherry. Images were captured digitally and analyzed using the imaging software: olympus fluoview v.4.2.

### ROS detection

To detect hydrogen peroxide (H_2_O_2_) or superoxide (O_2_^−^) by histochemical staining, Arabidopsis leaves and stalk tip tissues were collected and immersed in freshly prepared solutions of either 3,3′‐diaminobenzidine (DAB) (1 mg ml^−1^ DAB in Tris–HCl buffer (pH 3.8)) or nitroblue tetrazolium (NBT) (0.1% (w/v) NBT in 50 mM PB buffer (pH 7.5)). After incubation overnight in darkness at room temperature samples were destained in 95% (w/v) ethanol by heating in a boiling water‐bath to remove the Chl. Images were captured with a digital camera (Qiu *et al*., 2021).

### Aniline blue staining and PD permeability assays

*Nicotiana benthamiana* and *A. thaliana* leaves were infiltrated with 0.1% aniline blue (Sigma‐Aldrich) and incubated for 5 min in the dark. The infiltrated leaf tissue was dissected out, washed with sterile water, and then transferred to the confocal microscope for imaging.

Plasmodesmata permeability assays were performed essentially as described previously (Cui *et al*., 2015). One microliter of 1 mM 5(6)‐carboxyfluorescein diacetate (CFDA) was loaded on the adaxial leaf surface and incubated for 5 min, followed by removal of dye with sterile water and imaging of the abaxial leaf surface by confocal microscopy.

The ImageJ software (http://rsbweb.nih.gov/ij/) was employed for quantification of signals. The images were converted from color scale to grayscale. A plug‐in tool for semiautomated image analysis was used as described for the quantification of fluorescence intensity (Huang *et al*., 2022). For PD permeability assay, ‘Plot profile’ under the ‘Analyze’ menu in the ImageJ software was used as described (Cui *et al*., 2015).

### Statistical analysis

All experiments reported in this study were repeated at least three time except otherwise specified. Statistical differences between samples were determined by two‐tailed, unpaired Student's *t*‐test with equal variance (computed with Microsoft Excel) and sample differences were considered to be statistically significant if *P* < 0.05, indicated in figure legends as *, *P* < 0.05; **, *P* < 0.01; ***, *P* < 0.001.

## Results

### AtOSM34 and AtOLP are PD‐located proteins, and their expression is differentially regulated by TuMV infection

**Fig. 1:**
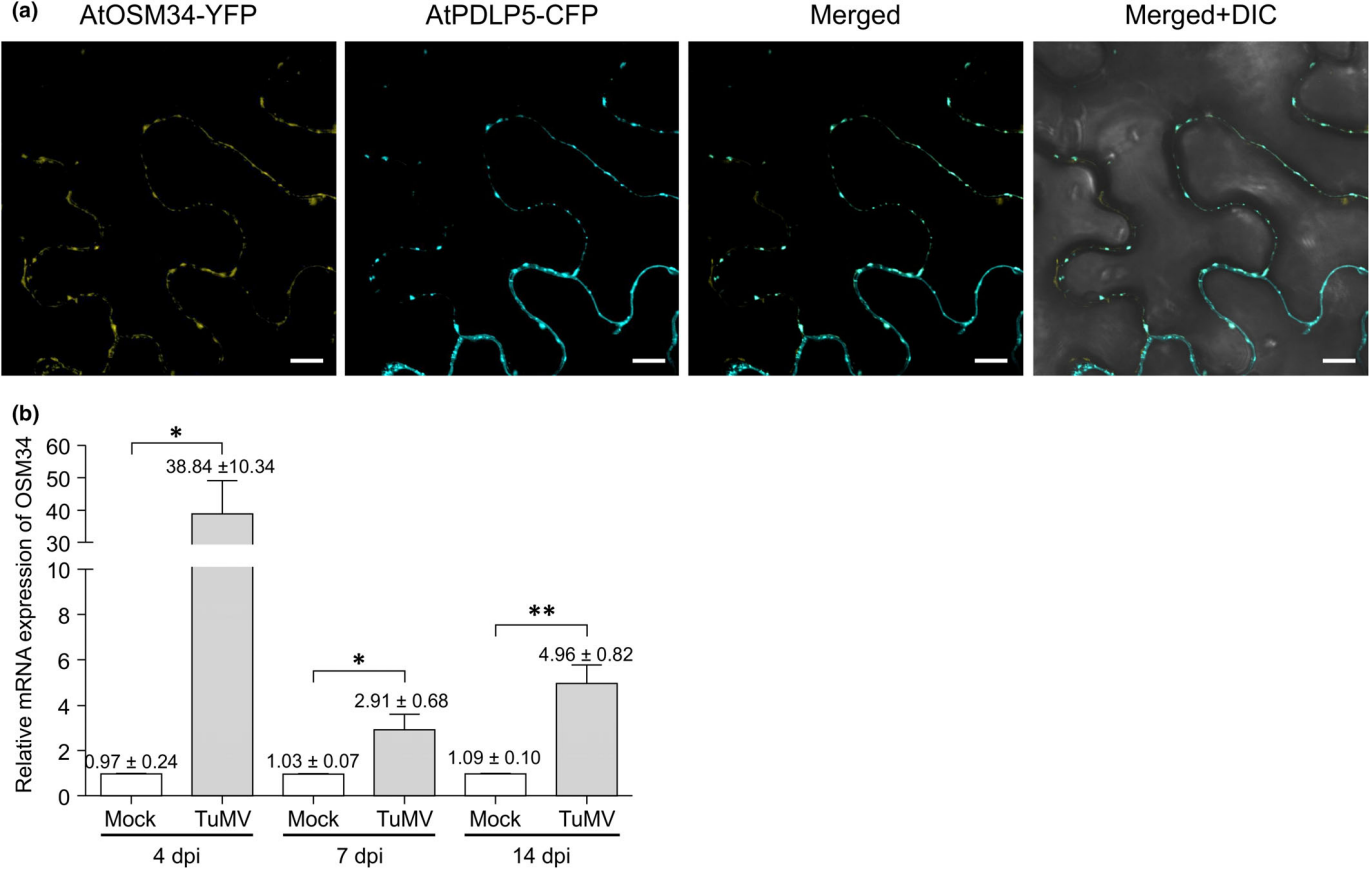
AtOSM34 is a PD‐located protein and *AtOSM34* is upregulated in TuMV‐infected plants. (a) Colocalization of AtOSM34‐YFP with the PD marker AtPDLP5‐CFP. Transient co‐expression of AtOSM34‐YFP and AtPDLP5‐CFP in *Nicotiana benthamiana* leaf cells. Images were taken at 48 h postinfiltration (hpi). DIC, differential interference contrast. Bar, 10 μm. (b) Relative mRNA expression levels of *AtOSM34* in the inoculated leaves at 4 d postinfiltration (dpi) and stalk tip tissues at 7 and 14 dpi from Arabidopsis plants inoculated with TuMV‐GFP, compared with that in corresponding tissues from mock‐inoculated control plants. Values are means ± SE (*n* = 3) and are presented as arbitrary units relative to mock. The experiment was repeated three times and each consisted of three biological replicates with each including a pooled sample from three plants. *AtActin II* transcripts in the same sample were used as an internal control. Statistically significant differences, determined by unpaired two‐tailed Student's *t*‐test comparing mock and TuMV‐inoculated plants are indicated: *, *P* < 0.05; **, *P* < 0.01.

In a recent quantitative, comparative proteomic study on the PD‐enriched fraction from *N. benthamiana* leaves, our lab identified 148 significantly differentially accumulated proteins including three OLPs (NbS00045440g0005.1, NbS00012471g0003.1 and NbS00007534g0005.1) in response to TuMV infection (Park *et al*., 2017). Compared with the protein level in the corresponding fraction from healthy control, the first OLP accumulated by > 70 fold in the PD‐enriched fraction from TuMV‐infected leaves, whereas each of the other two was reduced by *c*. 3.7 fold (Park *et al*., 2017). Blast (the basic local alignment search tool) searches against the Arabidopsis database identified At4G11650 (OSMOTIN 34, AtOSM34) to be the ortholog of NbS00045440g0005.1 (designated as NbOSM), and At2G28790 (AtOLP) as the ortholog of both NbS00012471g0003.1 (NbOLP1) and NbS00007534g0005.1 (NbOLP2) (Park *et al*., 2017). Since Arabidopsis is susceptible to TuMV infection and is an ideal model plant for molecular genetic studies, we used the Arabidopsis‐TuMV pathosystem to characterize the role of these OLPs in viral infection. An alignment of amino acid (aa) sequences using the Muscle algorithm (http://www.ebi.ac.uk/Tools/msa/muscle/) revealed that AtOSM34 shares 17.21% aa identity with AtOLP and 65.85% with NbOSM, and AtOLP shares 69.44% and 67.19% aa identity with NbOLP1 and NbOLP2, respectively (Fig. S1a). We cloned the coding cDNA sequences of AtOSM34, AtOLP, NbOSM, NbOLP1 and NbOLP2 and determined their subcellular localization. A YFP tag was fused to their N or C‐terminus and the fusion proteins were transiently expressed in *N. benthamiana* leaf cells via agroinfiltration. We found that the fusion proteins formed punctuates along the cell wall boundary, a typical PD distribution pattern (data not shown). We then did a colocalization experiment. AtOSM34‐YFP, AtOLP‐YFP, NbOSM‐YFP, NbOLP1‐YFP and NbOLP2‐YFP were transiently coexpressed with the PD protein marker AtPDLP5 (Thomas *et al*., 2008), which was tagged by a cyan fluorescent protein (AtPDLP5‐CFP). Confocal microscopy analyses revealed that AtOSM34‐YFP, AtOLP‐YFP, NbOSM‐YFP, NbOLP1‐YFP and NbOLP2‐YFP colocalized very well with AtPDLP5‐CFP along the cell wall (Figs 1a, S2a, S3a–c). These data confirm that AtOSM34, AtOLP and their orthologs in *N. benthamiana* are PD‐located proteins.

To determine if TuMV infection affects *AtOSM34* and *AtOLP* expression at the RNA level, reverse transcription quantitative polymerase chain reaction was conducted using total RNAs from both inoculated leaves (at 4 dpi) and stalk tip tissues (at 7 and 14 dpi) of mock and TuMV‐inoculated Arabidopsis. In TuMV‐inoculated leaves, the *AtOSM34* mRNA level increased by 40 fold (Fig. 1b). In comparison with mock‐treated control, significant higher expression levels were also found in stalk tip tissues of TuMV‐inoculated plants at 7 and 14 dpi. By contrast, *AtOLP* expression was downregulated by TuMV infection, particularly at 4 and 7 dpi (Fig. S2b). Consistent to the expression patterns of their orthologs in Arabidopsis, *NbOSM* in TuMV‐infected *N. benthamiana* was significantly upregulated (Fig. S3d), whereas *NbOLP1/2* was downregulated (Fig. S3e). These results are consistent with the proteomic data from TuMV‐infected *N. benthamiana* plants (Park *et al*., 2017). Together, these data suggest that Arabidopsis AtOSM34 and AtOLP and their orthologs (NbOSM and NbOLP1/2, respectively) in *N. benthamiana* are plasmodesmal proteins, and that *AtOSM34* and *NbOSM* are upregulated, while *AtOLP* and *NbOLP1/2* are downregulated in response to TuMV infection.

### Downregulation of AtOSM34 but not AtOLP inhibits TuMV infection

**Fig. 2:**
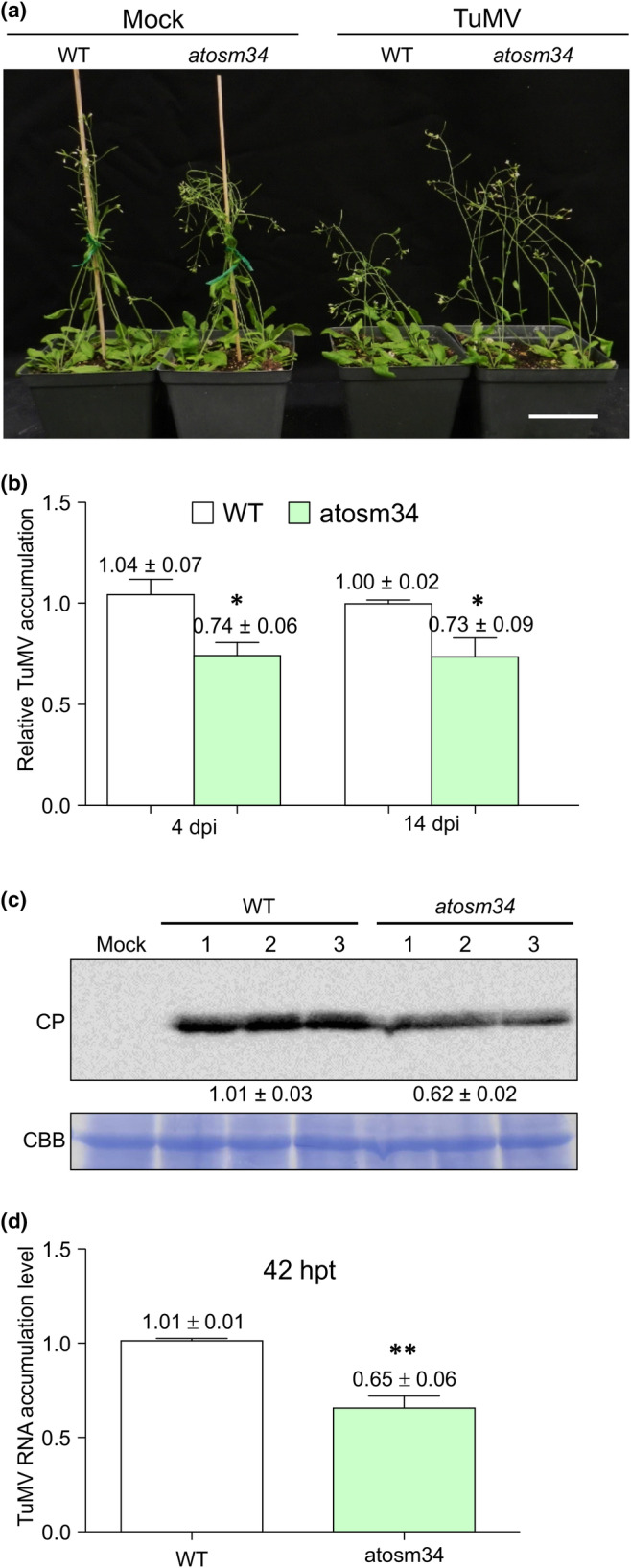
Knockdown of *AtOSM34* in Arabidopsis inhibits TuMV infection. (a) Phenotypes of wild‐type (WT) Col‐0 and *atosm34* mutant plants inoculated with TuMV‐GFP or mock‐inoculated at 15 d postinfiltration (dpi). Scale bar, 5 cm. (b) Reverse transcription quantitative polymerase chain reaction analysis of TuMV RNA accumulation levels in WT and *atosm34* plants at 4 and 14 dpi. Total RNA was extracted from inoculated leaves at 4 dpi and inflorescence tip tissues at 14 dpi. (c) Immunoblotting analysis of TuMV CP in WT and *atosm34* plants at 14 dpi. Total proteins were extracted from inflorescence tip tissues of WT and *atosm34* plants at 14 dpi and immunoblotted with anti‐TuMV CP antibodies. Values underneath the blot indicate the relative TuMV CP signals quantified by ImageJ software (mean ± SD; *n* = 3). The Coomassie Brilliant Blue R‐250‐stained Rubisco large subunit (CBB) serves as a loading control. (d) TuMV transfection assay with protoplasts isolated from WT and *atosm34* mutant plants. TuMV RNA was isolated from protoplasts transfected by TuMV‐GFP at 42 h posttransfection (hpt). In (b, d), values are means ± SE (*n* = 3) and are presented as arbitrary units relative to TuMV‐inoculated WT. Both experiments were repeated three times and each consisted of three biological replicates with each including a pooled sample from three plants. *AtActin II* transcripts in the same sample were used as an internal control. Statistically significant differences, determined by unpaired two‐tailed Student's *t*‐test comparing TuMV‐inoculated WT and *atosm34* plants are indicated: *, *P* < 0.05; **, *P* < 0.01.

To examine whether AtOSM34 and AtOLP are involved in TuMV infection, we obtained *AtOSM34* and *AtOLP* transfer DNA (T‐DNA) mutants from the ABRC. The mutant line CS916067 was identified to be a homozygous *AtOSM34*‐knockdown mutant (Fig. S4a,b). CS819509 and CS850431 were confirmed to be *AtOLP*‐knockdown and knockout mutants, respectively (Fig. S5a,b). Gene expression of *AtOSM34* and *AtOLP* in these mutants was verified by reverse transcription polymerase chain reaction and/or reverse transcription quantitative polymerase chain reaction (Figs S4c,d, S5b). These mutants did not show distinguishable morphological difference from the wild‐type (WT) Col‐0 plant under normal growth conditions (Figs S4e, S5c). After inoculation with TuMV, *atolp* mutant plants produced similar typical viral symptoms with WT Arabidopsis (Fig. S5d), whereas the mutant *atosm34* showed much milder symptoms (Fig. 2a). Consistently, no significant difference was found between TuMV genomic RNA levels in WT and *atolp* mutant plants at 14 dpi (Fig. S5e), whereas TuMV RNA abundance was significantly reduced in the *atosm34* mutant, compared to that in WT plants (Fig. 2b). Immunoblotting confirmed that the CP (coat protein) level was also significantly reduced in the *atosm34* mutant (Fig. 2c). We further carried out an Arabidopsis protoplast transfection assay. TuMV plasmid pCambiaTuMV::GFP (Cotton *et al*., 2009) was transfected into protoplasts isolated from WT, *atosm34* and *atolp* plants, followed by reverse transcription quantitative polymerase chain reaction to quantify viral RNA accumulation. Viral RNA accumulation was significantly reduced in protoplasts isolated from the *atosm34* mutant at 42 hpt (Fig. 2d), and no significant difference was found between viral RNA levels in protoplasts from WT and *atolp* mutant lines (Fig. S5h). Taken together, these data suggest that downregulation of AtOSM34 but not AtOLP suppresses TuMV symptom development and viral RNA accumulation in Arabidopsis plants and protoplasts.

**Fig. 3:**
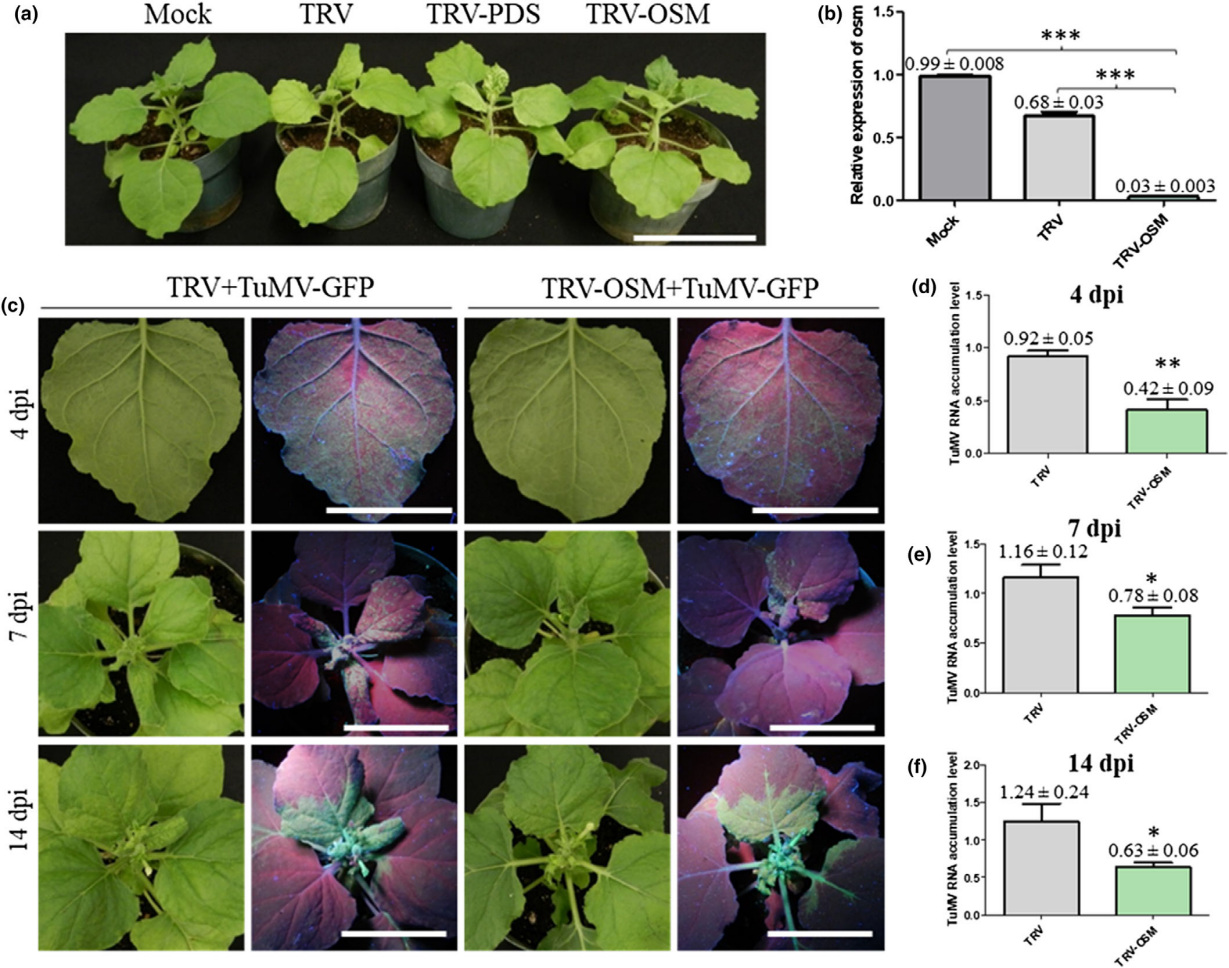
Knockdown of *NbOSM* inhibits TuMV infection in *Nicotiana benthamiana*. (a) Phenotypes of *N. benthamiana* plants agroinfiltrated with buffer (mock), TRV empty vector (TRV), TRV‐PDS and TRV‐OSM. Images were taken at 10 d postinfiltration (dpi). Scale bar, 10 cm. (b) Quantification of *NbOSM* gene expression by reverse transcription quantitative polymerase chain reaction. (c) Phenotypes of *N. benthamiana* plants pretreated with TRV empty vector (TRV), and TRV‐OSM plants, followed by infiltration with TuMV‐GFP. Representative images were taken at 4, 7 and 14 dpi under normal light and UV illumination. Scale bar, 5 cm. (d–f) The effect of knockdown of *NbOSM* on TuMV RNA accumulation. *Nicotiana benthamiana* plants were pretreated with TRV empty vector (TRV) and TRV‐OSM, followed by infiltration with TuMV‐GFP. Inoculated leaves at 4 dpi (d), and systemically infected leaves at 7 dpi (e) and 14 dpi (f) were collected for RNA purification, and viral RNA was quantified by reverse transcription quantitative polymerase chain reaction analysis of the CP RNA level. In (b, d–f), values are means ± SE (*n* = 3) and are presented as arbitrary units relative to mock in (b) or to TRV‐treated samples in (d–f). The experiment consisted of three biological replicates and each included a pooled sample from three plants. *NbActin* transcripts in the same sample were used as an internal control. All experiments were repeated three times. Statistically significant differences, determined by unpaired two‐tailed Student's *t*‐test are indicated: *, *P* < 0.05; **, *P* < 0.01; ***, *P* < 0.001.

To test whether downregulation of the ortholog of AtOSM34 in *N. benthamiana* inhibits TuMV infection, we knocked down the expression of *NbOSM* using a TRV‐based VIGS approach (Senthil‐Kumar & Mysore, 2014; Park *et al*., 2017). The recombinant TRV vectors TRV‐OSM and TRV‐PDS could efficiently silence *OSM* and *PDS* (*phytoene desaturase*) genes in *N. benthamiana* (Fig. 3a,b). In the *NbOSM*‐knockdown *N. benthamiana* plants, TuMV infection was inhibited (Fig. 3c) and viral RNA accumulation was significantly reduced at all the three time points tested (Fig. 3d–f). These data suggest that downregulation of *NbOSM* in *N. benthamiana* also inhibits TuMV infection.

### Overexpression of AtOSM34 promotes TuMV infection and viral accumulation

To assess if overexpression of AtOSM34 and AtOLP affects TuMV infection, we generated transgenic Arabidopsis lines expressing AtOSM34‐Flag‐4×Myc and AtOLP‐Flag‐4×Myc. Their expression in transgenic plants was confirmed by reverse transcription quantitative polymerase chain reaction (Figs S4f, S5b). The AtOSM34 or AtOLP overexpression lines were morphologically similar with WT plants (Figs S4g, S5c).

**Fig. 4:**
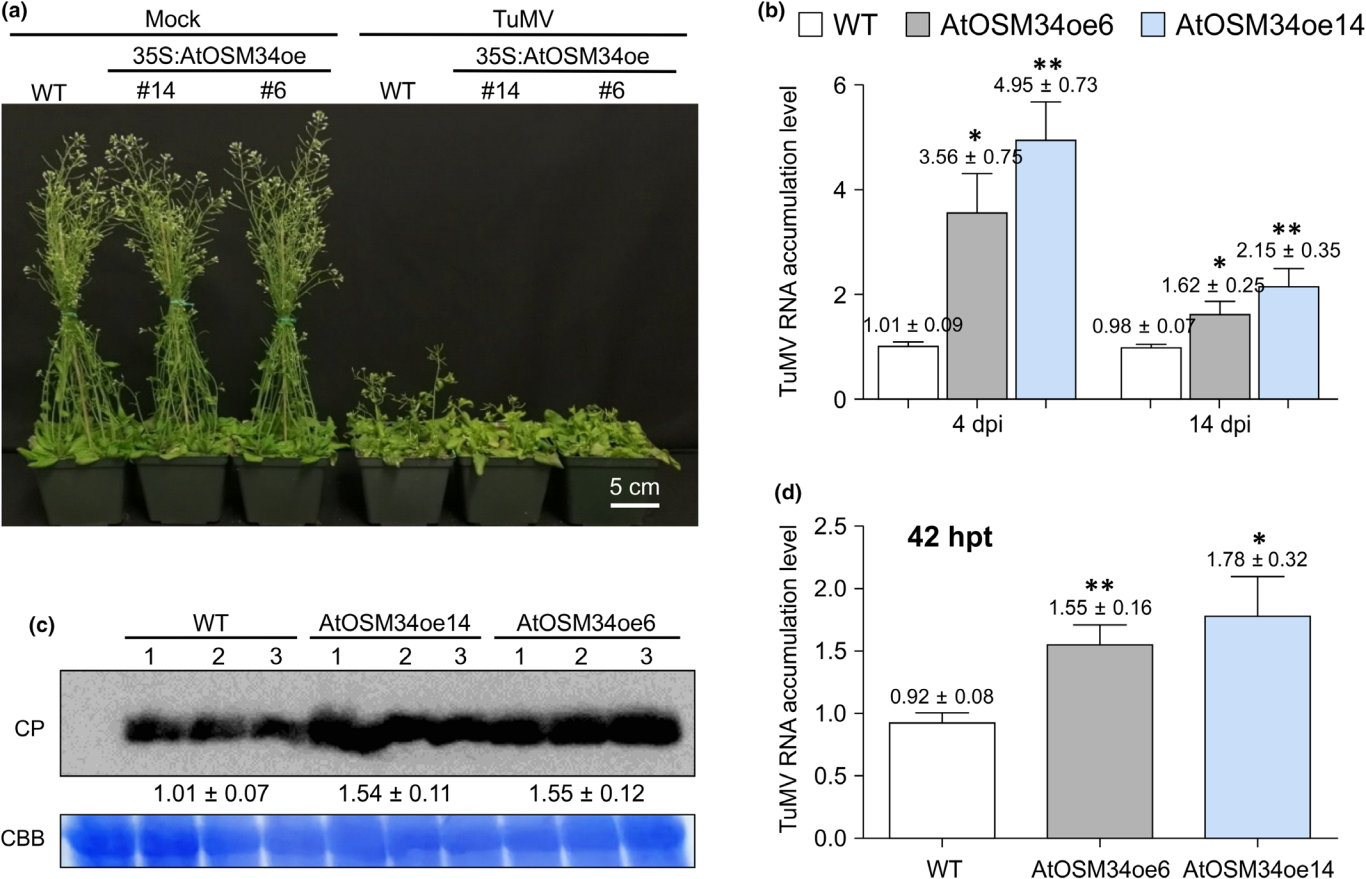
Overexpression of AtOSM34 promotes TuMV infection. (a) Symptom comparison between TuMV‐infected WT and AtOSM34 transgenic overexpression Col‐0 Arabidopsis plants at 19 d postinfiltration (dpi). Mock, inoculated with buffer; TuMV, inoculated with TuMV. (b) Reverse transcription quantitative polymerase chain reaction analysis of TuMV RNA level in WT and AtOSM34 transgenic overexpression Arabidopsis plants at 4 and 14 dpi. (c) Immunoblotting analysis of TuMV CP levels in WT and AtOSM34 transgenic overexpression Arabidopsis lines at 14 dpi. Total proteins were extracted from inflorescence tip tissues of TuMV‐infected or mock‐treated plants at 14 dpi and immunoblotted with anti‐TuMV CP antibodies. Values subtending the blot indicate the relative TuMV CP signals quantified by ImageJ software. The Coomassie Brilliant Blue R‐250‐stained Rubisco large subunit (CBB) serves as a loading control. (d) TuMV transfection assay of protoplasts isolated from WT and AtOSM34 transgenic overexpression Arabidopsis leaves. Total RNA was isolated from the protoplasts transfected by TuMV‐GFP at 42 h posttransfection (hpt). Viral RNA was quantified by reverse transcription quantitative polymerase chain reaction with primers specific for the CP coding region. In (b, d), values are means ± SE (*n* = 3) and are presented as arbitrary units relative to TuMV‐inoculated WT. The experiment was repeated three times and each consisted of three biological replicates with each including a pooled sample from three plants. *AtActin II* transcripts in the same sample were used as an internal control. Statistically significant differences, determined by unpaired two‐tailed Student's *t*‐test comparing TuMV‐inoculated WT and AtOSMoe6 or AtOSM34oe14 overexpression plants are indicated: *, *P* < 0.05; **, *P* < 0.01.

Two representative transgenic lines of the AtOSM34‐Flag‐4×Myc and AtOLP‐Flag‐4×Myc overexpression plants were inoculated with TuMV. In comparison with the WT plants, the transgenic AtOSM34 overexpression lines were more susceptible to TuMV infection, showing more severe symptoms (Fig. 4a). Higher levels of the viral genomic RNA and CP were detected in TuMV‐infected AtOSM34 overexpression plants (Fig. 4b,c). We also performed a protoplast transfection assay to observe the effect of overexpressing AtOSM34 on viral RNA accumulation. Protoplasts were isolated from WT Arabidopsis and AtOSM34 overexpression lines, and transfected with pCambiaTuMV::GFP, followed by reverse transcription quantitative polymerase chain reaction to quantify viral RNA accumulation at 42 hpt. The TuMV viral RNA level was significantly higher in the protoplasts isolated from AtOSM34 overexpression transgenic lines, compared with that in control protoplasts (Fig. 4d). By contrast, transgenic AtOLP overexpression lines developed similar viral symptoms with the WT plants (Fig. S5f). No significant difference was found between the viral RNA levels in WT and AtOLP overexpression plants (Fig. S5g). Consistently, similar viral RNA accumulation levels were observed in TuMV transfected protoplasts from the AtOLP overexpression and WT plants (Fig. S5i). These data show that the overexpression of AtOSM34 rather than AtOLP promotes TuMV infection.

### Transient overexpression of AtOSM34 facilitates TuMV intercellular movement and viral accumulation

**Fig. 5:**
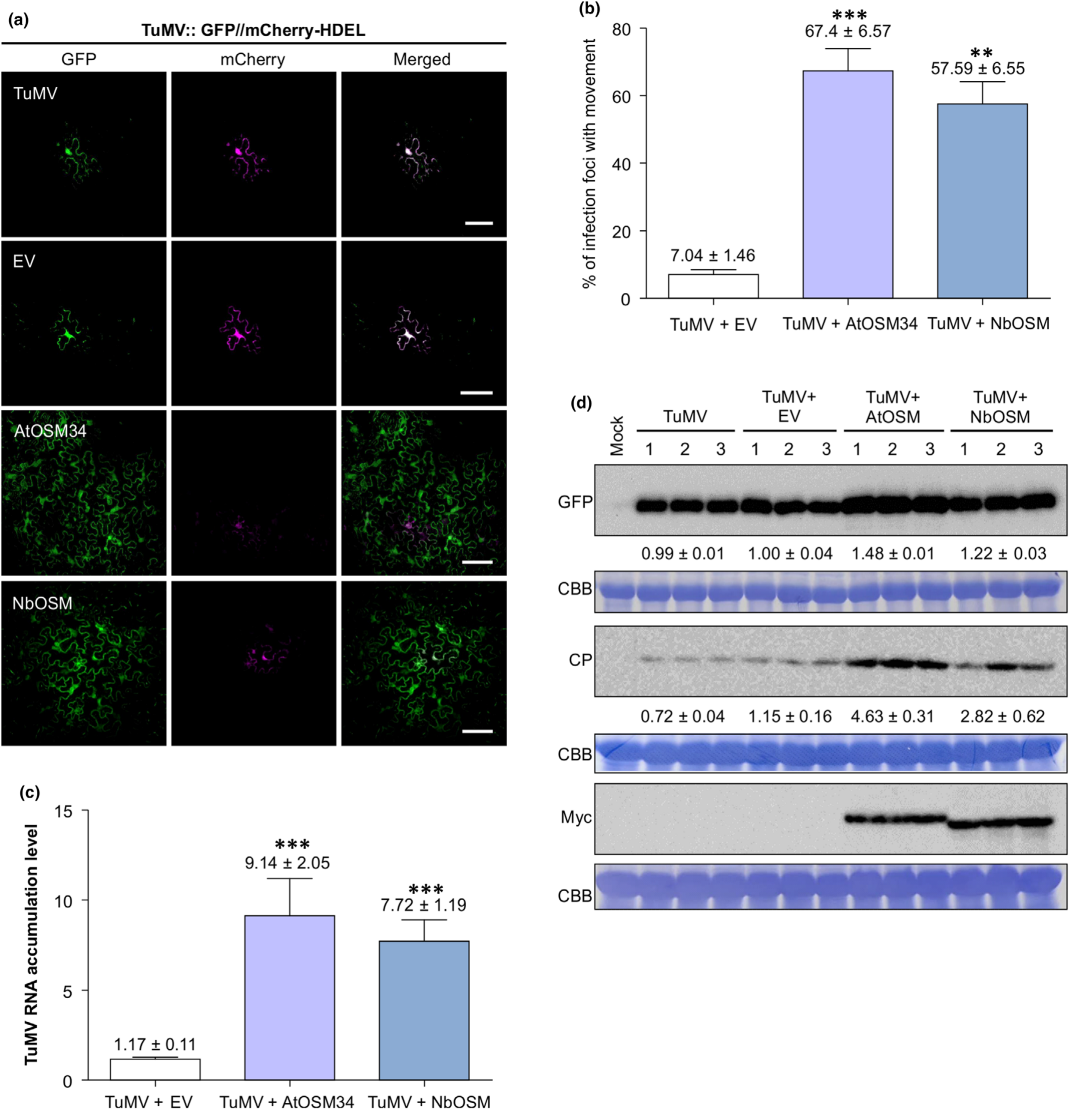
Transient overexpression of AtOSM34 facilitates TuMV intercellular movement. (a) Confocal microscopy observation of viral intercellular movement in *Nicotiana benthamiana* leaf tissues. *Agrobacterium* cells harboring the plasmid TuMV::GFP//mCherry‐HDEL (OD_600_ = 0.001) were coinfiltrated into leaves of *N. benthamiana* with either buffer, empty vector (EV), AtOSM34‐Flag‐4×Myc, or NbOSM. Red fluorescence emitted by mCherry indicates the mCherry‐HDEL expression cassette product, and green fluorescence by GFP indicates the recombinant TuMV‐GFP genome product. Cells emitting both red and green fluorescence represent primarily infected ones whereas those emitting GFP only are secondarily infected cells. Images were taken at 66 h postinfiltration (hpi). Bar, 100 μm. (b) Statistical analysis of cell‐to‐cell movement frequency in (a). This experiment was repeated three times. In each experiment, 10 plants were used for each plasmid combination and 10 infection foci per plant were observed for data analyses. Values are means with SE. Statistically significant differences, determined by unpaired two‐tailed Student's *t*‐test comparing the sample treated with TuMV + EV with that with TuMV + AtOSM34 or TuMV + NbOSM are indicated: **, *P* < 0.01; ***, *P* < 0.001. (c) Reverse transcription quantitative polymerase chain reaction analysis of TuMV genomic RNA accumulation in *N. benthamiana* plants at 68 hpi. Total RNA was extracted from leaves of *N. benthamiana* infiltrated with TuMV::GFP//mCherry‐HDEL and empty vector (TuMV + EV), AtOSM34‐Flag‐4×Myc (TuMV + AtOSM34) or NbOSM (TuMV + NbOSM). Data are means from three independent experiments with SE and are presented as arbitrary units relative to the TuMV + EV sample. *NbActin* transcripts in the same sample were used as an internal control. Statistically significant differences, determined by unpaired two‐tailed Student's *t*‐test comparing the sample treated with TuMV + EV with that treated with TuMV + AtOSM34 or TuMV + NbOSM are indicated: ***, *P* < 0.001. (d) Immunoblotting analysis of viral CP and GFP protein accumulation in the infiltrated leaf tissues from *N. benthamiana* plants at 68 hpi. Total proteins were extracted from the infiltrated leaf tissues at 68 hpi, and immunoblotted with anti‐GFP, anti‐TuMV CP, and anti‐Myc antibodies, respectively. Values underneath the blots indicate the relative TuMV CP signals quantified by ImageJ software (mean ± SD; *n* = 3). The Coomassie Brilliant Blue R‐250‐stained Rubisco large subunit (CBB) serves as a loading control.

As AtOSM34 and AtOLP are PD‐located proteins, we speculated that they might be involved in viral cell‐to‐cell movement. To test this possibility, we co‐agroinfiltrated a double‐cassette expression vector TuMV‐GFP//mCherry‐HDEL (Dai *et al*., 2020) and each of the OLP expression vectors into *N. benthamiana* leaf tissues. The double‐cassette expression vector contains two expression cassettes: one for the transcription of the TuMV full‐length cDNA infectious clone tagged by GFP and the other for the expression of the red fluorescent protein mCherry. When this double‐cassette vector is agroinfiltrated into plant leaf cells, the primarily infected cell will emit both green and red fluorescence, while the secondarily infected cells will only radiate green fluorescence as a result of viral intercellular movement (Dai *et al*., 2020). Potyviral intercellular movement usually starts at 72–96 h postinfiltration (hpi) (Dai *et al*., 2020). Based on this, we monitored viral movement using confocal microscopy. Indeed, at 66 hpi, no cell‐to‐cell movement was visible in the control samples (Fig. 5a). By contrast, the viral intercellular movement was obvious in leaves transiently expressing AtOSM34 or NbOSM (Fig. 5a,b).

We further conducted reverse transcription quantitative polymerase chain reaction and/or immunoblotting analysis to assess viral infection in coinfiltrated leaves. A significant increase in TuMV genomic RNA, CP and GFP (derived from the recombinant TuMV) was detected in plants coinfiltrated with AtOSM34 or NbOSM, compared with control plants (Fig. 5c,d). Interestingly, the transient overexpression of AtOLP, NbOLP1 or NbOLP2 could also enhance viral intercellular movement (Fig. S6a), but did not significantly affect GFP accumulation (Fig. S6b).

### PD permeability and callose deposition are altered in AtOSM34 or AtOLP overexpression plants

**Fig. 6:**
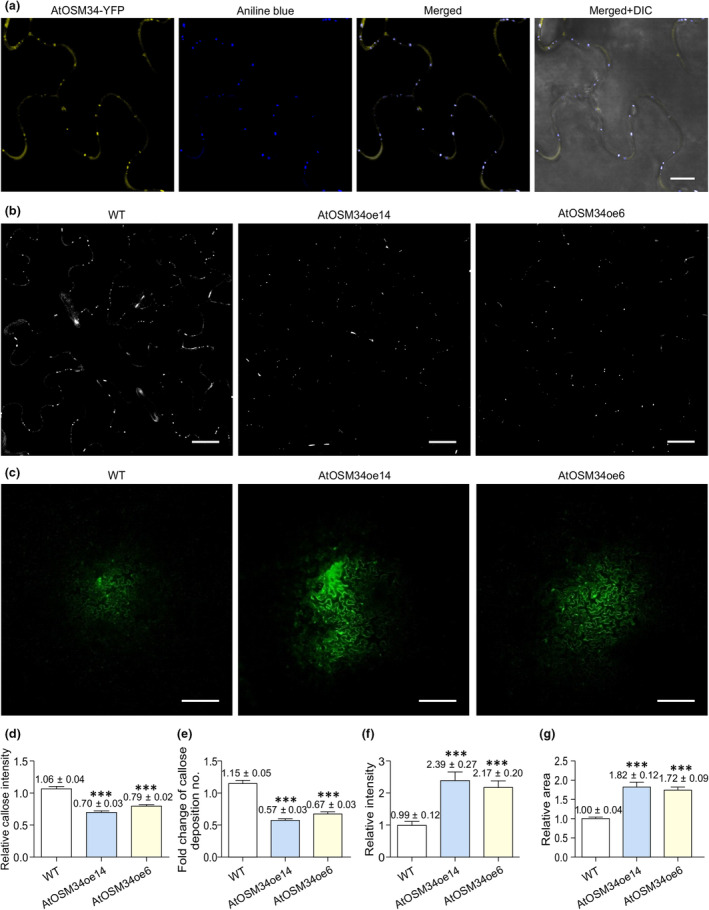
Overexpression of AtOSM34 reduces PD callose deposition and increases PD permeability. (a) AtOSM34‐YFP was transiently expressed in *Nicotiana benthamiana* leaves via agroinfiltration, then infiltrated with aniline blue at 48 h postinfiltration (hpi) and incubated for 5 min before imaging. Images were taken at 48 hpi. DIC, differential interference contrast. Bar, 10 μm. (b, d, e) Comparison of callose deposition levels in WT and transgenic AtOSM4 overexpression Arabidopsis leaf tissues by aniline blue staining. Two representative transgenic lines, AtOSM34oe14 and AtOSMoe6 were used in this experiment. (b) Confocal images of callose staining. Bar, 20 μm. (d) Relative callose deposition intensity. (e) Quantification of fold change of callose deposition number. (c, f, g) Comparison of PD permeability of WT and transgenic AtOSM34 overexpression Arabidopsis leaves by CFDA‐based DANS dye loading assay. (c) Confocal images of CFDA movement. Bar, 200 μm. (f) Relative fluorescence intensity of CFDA. (g) Relative diffusion areas of CFDA. In (d–g), values are means with SE from three independent experiments. In each experiment, 10 plants were used for each treatment. Statistically significant differences, determined by unpaired two‐tailed Student's *t*‐test comparing WT and overexpression plants are indicated: ***, *P* < 0.001.

Since TuMV intercellular movement through PD was enhanced in plants overexpressing AtOSM34 (Fig. 5a,b) or AtOLP (Fig. S6a), we wondered whether their overexpression affects PD permeability. The deposition of callose, a linear β‐1,3‐glucan polysaccharide, at the PD neck region dynamically regulates PD permeability (Petit *et al*., 2020). Aniline blue is a fluorescent dye that specifically stains callose, which has also been widely used as a plasmodesmal marker (Northcote *et al*., 1989; Thomas *et al*., 2008). We stained *N. benthamiana* leaf tissues agroinfiltrated with the AtOSM34‐YFP or AtOLP‐YFP expression vector using aniline blue, and confirmed that the aniline blue‐stained callose colocalized with PD labelled by these YFP‐labelled OLPs (Figs 6a, S2a). Then, we conducted an aniline blue staining assay to observe the effect of overexpressing AtOSM34 or AtOLP on PD callose deposition. Callose deposition was quantified by ImageJ. Compared with that in WT, the PD callose deposition in transgenic plants overexpressing AtOSM34 or AtOLP was lower (Figs 6b,d,e, S7a,c,d). Next, we performed a CFDA‐based Drop‐ANd‐See (DANS) assay (Cui *et al*., 2015; Sankoh & Burch‐Smith, 2021) to compare PD permeability in WT and transgenic AtOSM34 or AtOLP overexpression plants. A significant increase in PD permeability was found in the AtOSM34 or AtOLP overexpression lines (Figs 6c,f,g, S7b,e,f).

### AtOSM34 domains DII and DIII interact with TuMV 6K2

**Fig. 7:**
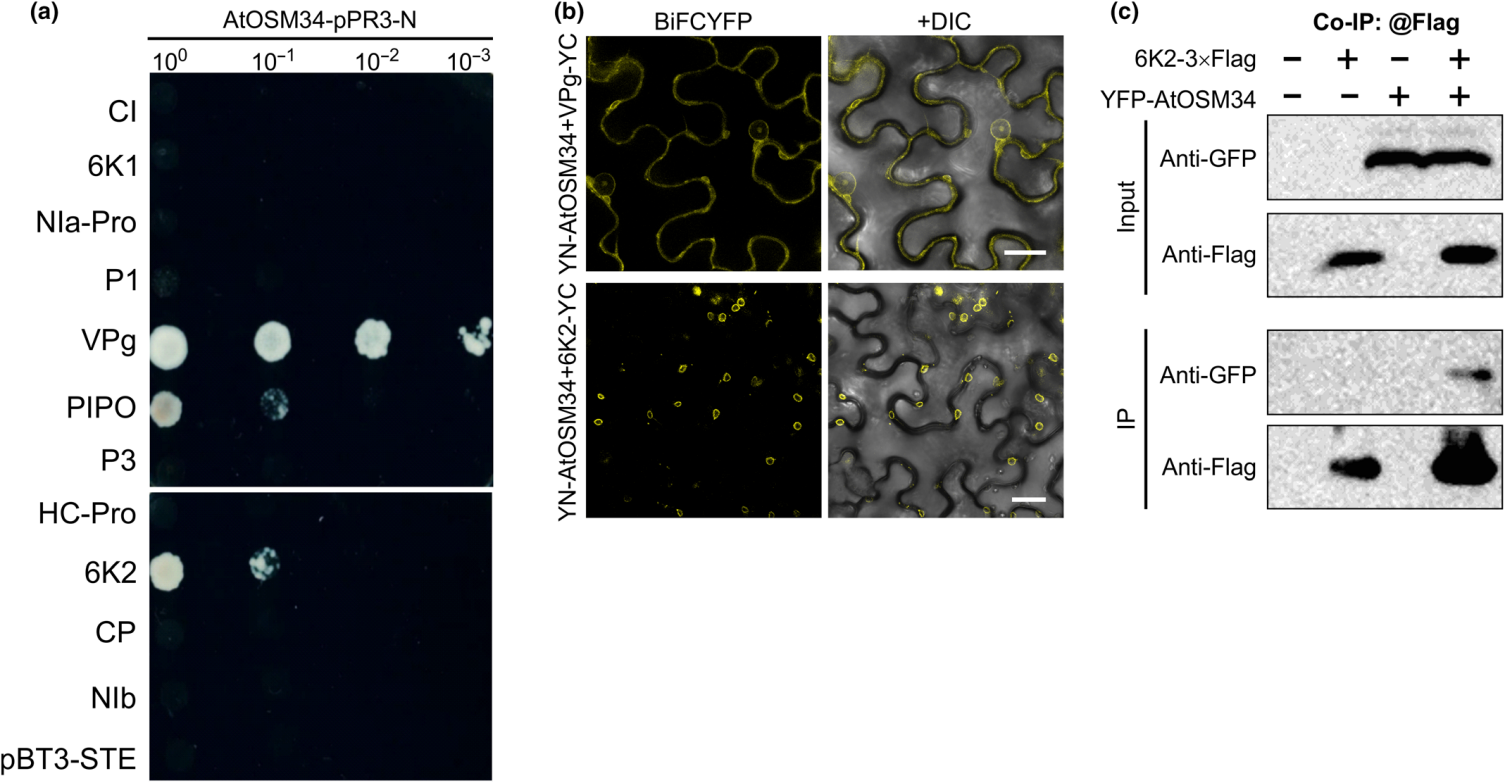
AtOSM34 interacts with TuMV proteins. (a) Screening for protein–protein interactions between AtOSM34 and each of the 11 TuMV viral proteins by the mYTH assay. Cotransformation of pBT3‐STE empty vector with AtOSM34‐pPR3‐N serves as a negative control. (b) Positive interactions of AtOSM34 with VPg and 6K2 confirmed by the BiFC assay. Yellow fluorescence was observed in *N. benthamiana* leaves coinfiltrated with YN‐AtOSM34/VPg‐YC or YN‐AtOSM34/6K2‐YC at 48 h postinfiltration (hpi). Confocal images were taken at 48 hpi. Bar, 20 μm. (c) Confirmation of the AtOSM34 and 6K2 interaction by the Co‐IP assay. Protein extracts were incubated with anti‐Flag M2 beads. Samples before (Input) and after (IP) immunoprecipitation were analyzed by immunoblotting with anti‐GFP or anti‐Flag antibodies.

To explore possible molecular mechanisms(s) underlying the proviral role of AtOSM34 in TuMV infection, we ascertained whether AtOSM34 interacts with any TuMV‐encoded proteins. Because AtOSM34 is a membrane‐associated protein, we used the mYTH system to screen for the TuMV proteins that interact with AtOSM34. Among 11 TuMV proteins, three including PIPO, 6K2, and VPg showed positive interactions with AtOSM34 by the mYTH assay (Fig. 7a). We then conducted the BiFC assay to confirm these interactions in *N. benthamiana* leaf cells. Positive interaction signals were detected in the leaf cells coexpressing AtOSM34 with 6K2 or VPg (Fig. 7b). No positive interaction was found between AtOSM34 and PIPO in *N. benthamiana* (Fig. S8a), suggesting the interaction of AtOSM34 with PIPO detected in Y2H is either very weak or artificial. No interaction signals were observed in the leaf tissues coexpressing YN‐AtOLP and 6K2‐YC or YN‐AtOLP and VPg‐YC (Fig. S8a). We further conducted Co‐IP assays to confirm the two combinations showing positive interactions. TuMV 6K2 and VPg, fused with the 3 × Flag tag, and the YFP‐AtOSM34 fusion protein were transiently coexpressed in *N. benthamiana* leaves. Our Co‐IP data validated the interactions of AtOSM34 with TuMV 6K2 (Fig. 7c), but not with TuMV VPg (Fig. S8c).

**Fig. 8:**
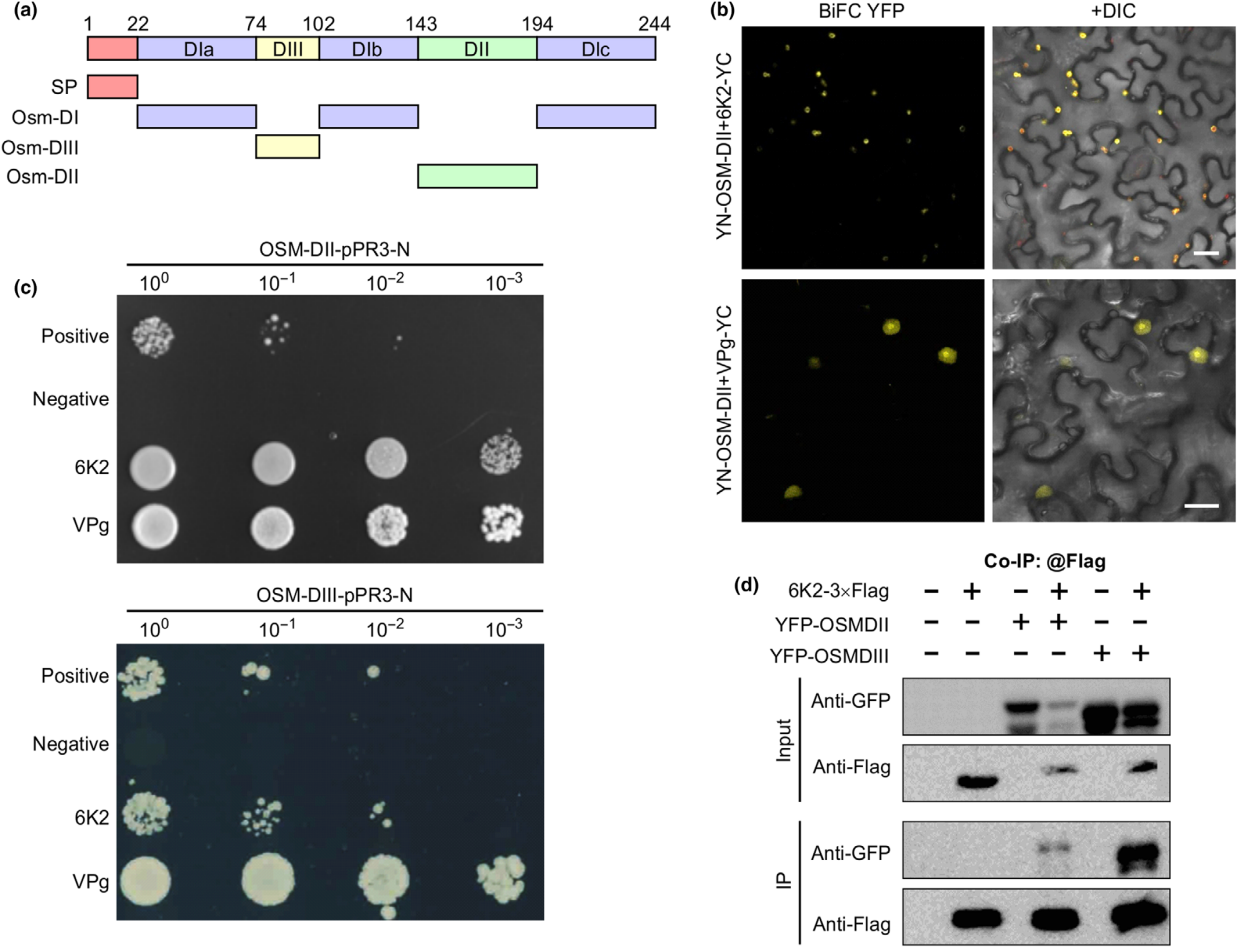
AtOSM34 domains DII and DIII interact with TuMV 6K2. (a) Diagrammatic representation of the AtOSM34 protein with conserved domains. The thick lines represent the location and length of predicted domains. Numbers indicate amino acid positions. (b) AtOSM34 DII interacts with 6K2 and VPg. The BiFC assay was conducted in *N. benthamiana* leaf cells. Confocal images were taken at 48 h postinfiltration (hpi). Bar, 20 μm. (c) mYTH assay of the interactions of DII and DIII of AtOSM34 with TuMV 6K2 and VPg in *S. cerevisiae* cells. (d) Co‐IP assay of the interactions of DII and DIII of AtOSM34 with TuMV 6K2. Protein extracts were incubated with anti‐Flag M2 beads. Samples before (Input) and after (IP) immunoprecipitation were analyzed by immunoblotting with anti‐GFP or anti‐Flag antibodies.

To map the domains of AtOSM34 that bind to TuMV 6K2, we performed a computer‐assisted prediction of the structure via Phyre 2 (http://www.sbg.bio.ic.ac.uk/phyre2). We found that AtOSM34 contains four domains including the SP (signal peptide), DI (domain I), DII (domain II) and DIII (domain III) (Fig. 8a). We constructed plant and yeast expression vectors to express these domains, and then used them for BiFC and mYTH assays. We also included VPg in these assays. The BiFC assay identified DII that interacted with 6K2 and VPg in *N. benthamiana* leaf cells (Fig. 8b). The mYTH assay confirmed these two positive interactions and also showed DIII that could bind to 6K2 and VPg in yeast cells (Fig. 8c). No interactions were detected between other domains and two viral proteins (Fig. S9a–d). Our co‐IP assay further validated the interaction of DII and DIII of AtOSM34 with 6K2 (Fig. 8d) but not with VPg (Fig. S9e). These results suggest that AtOSM34 interacts with TuMV 6K2 likely via its DII and DIII.

Since AtOSM34 DII showered a very strong interaction with 6K2, we investigated the effect of deletion of this domain on TuMV cell‐to‐cell movement and viral RNA accumulation. We found that transient expression of the DII deletion mutant, AtOSM34delDII, could still promote viral intercellular movement at 66 hpi (Fig. S10a), but to a much lesser extent, compared to AtOSM34. At 68 hpi, the mutant could also promote TuMV RNA accumulation by over fourfold (Fig. S10b). Compared to a ninefold increasement by AtOSM34, the DII deletion mutant lost > 60% of that capacity.

### AtOSM34 colocalizes with TuMV VRCs

**Fig. 9:**
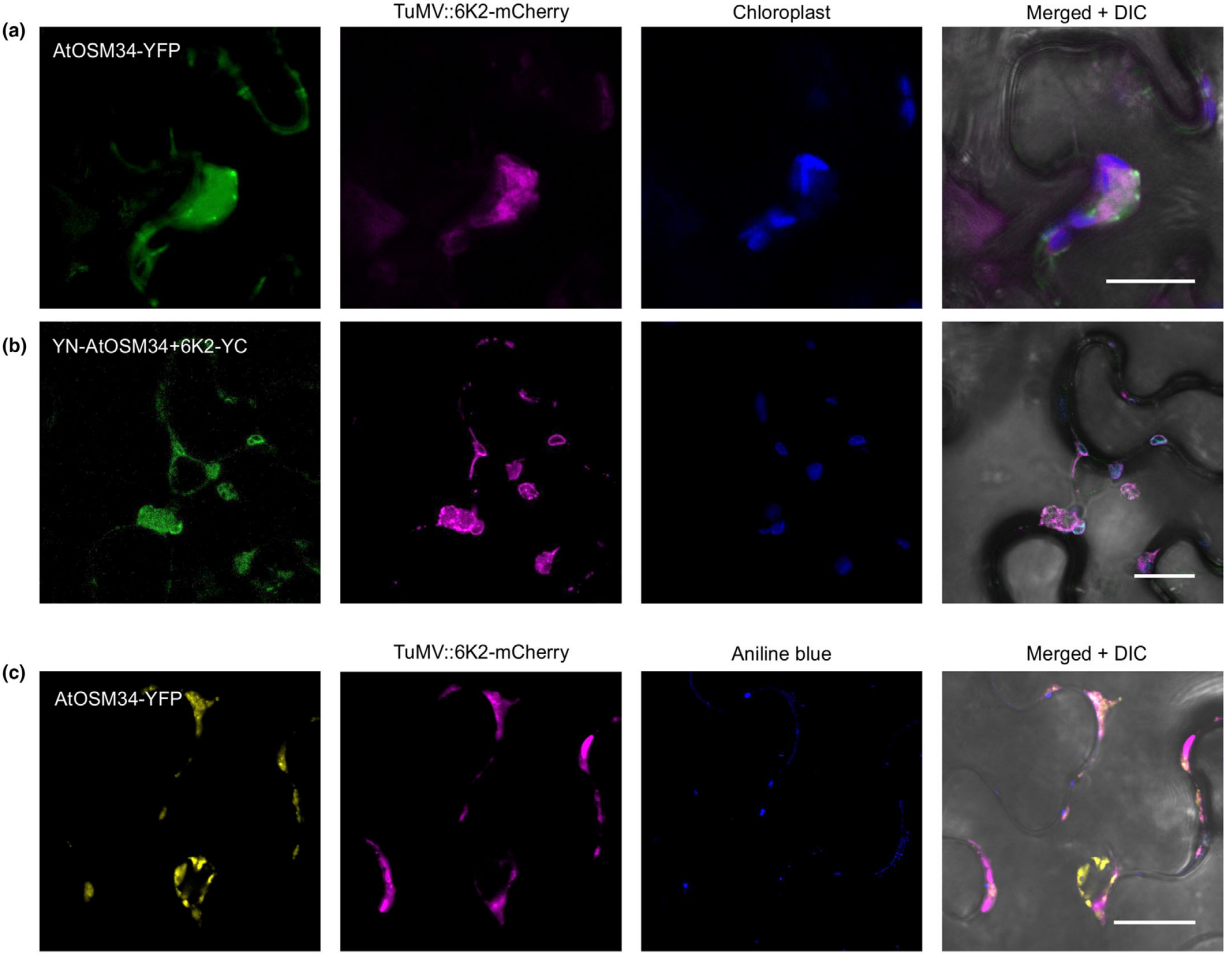
AtOSM34 or the AtOSM34‐6K2 interaction complex colocalizes with the TuMV VRC in *Nicotiana benthamiana* cells. (a) Colocalization of AtOSM34 with 6K2‐induced TuMV vesicles. AtOSM34‐YFP was coexpressed with the recombinant TuMV infectious clone TuMV::6K2‐mCherry in *N. benthamiana* leaf tissues and observed under a confocal microscope at 3 d postinfiltration (dpi). (b) Colocalization of the TuMV VRC labelled by mCherry with the YFP signals resulting from the interaction between 6K2 with AtOSM34. The infectious clone TuMV::6K2‐mCherry and BiFC expression vectors YN‐AtOSM34 and 6K2‐YC were coinfiltrated into epidemic cells of *N. benthamiana* leaves and the infiltrated leaf tissues were observed under a confocal microscope at 3 dpi. (c) Colocalization of AtOSM34 with 6K2‐induced TuMV vesicles to aniline blue staining. The leaf tissues transiently expressing AtOSM34‐YFP and TuMV::6K2‐mCherry were infiltrated with aniline blue at 3 dpi and incubated for 5 min before imaging. In (a–c): all images were taken at 3 dpi. Chloroplast, chloroplast autofluorescence; DIC, differential interference contrast. Bar, 20 μm.

To investigate whether AtOSM34 is recruited by TuMV VRC, AtOSM34 was expressed in *N. benthamiana* leaf cells infected with the recombinant TuMV infectious clone pCambiaTuMV::6K2‐mCherry (TuMV‐6K2‐mCherry). This recombinant full‐length cDNA infectious clone can produce an additional copy of 6K2 tagged by the red fluorescent protein mCherry (Cotton *et al*., 2009). Previous studies have established that the potyviral 6K2 protein induces the formation of ER‐derived vesicles that house the VRC for potyviral replication (Wei & Wang, 2008; Cotton *et al*., 2009) and many of these 6K2 vesicles traffic to and become associated with chloroplasts for robust viral replication (Wei *et al*., 2010a). Confocal microscopy analysis revealed that AtOSM34 colocalized with the chloroplast‐associated VRCs highlighted by 6K2‐mCherry in TuMV‐infected leaf cells (Fig. 9a).

Since AtOSM34 interacts with 6K2, we suspected that AtOSM34 might be recruited to the VRC by 6K2. The infectious clone TuMV‐6K2‐mCherry and two BiFC vectors YN‐AtOSM34 and 6K2‐YC were co‐agroinfiltrated into *N. benthamiana* leaf epidermal cells. The 6K2 and AtOSM34 interaction complex was found to colocalize with the VRCs (Fig. 9b), supporting the assumption that AtOSM34 may be recruited to the VRC via the interaction with 6K2. Some of these AtOSM34–VRC interaction complexes were in proximity to PD stained by callose binding dye aniline blue (Fig. 9c).

### AtOSM34 inhibits ROS burst

**Fig. 10:**
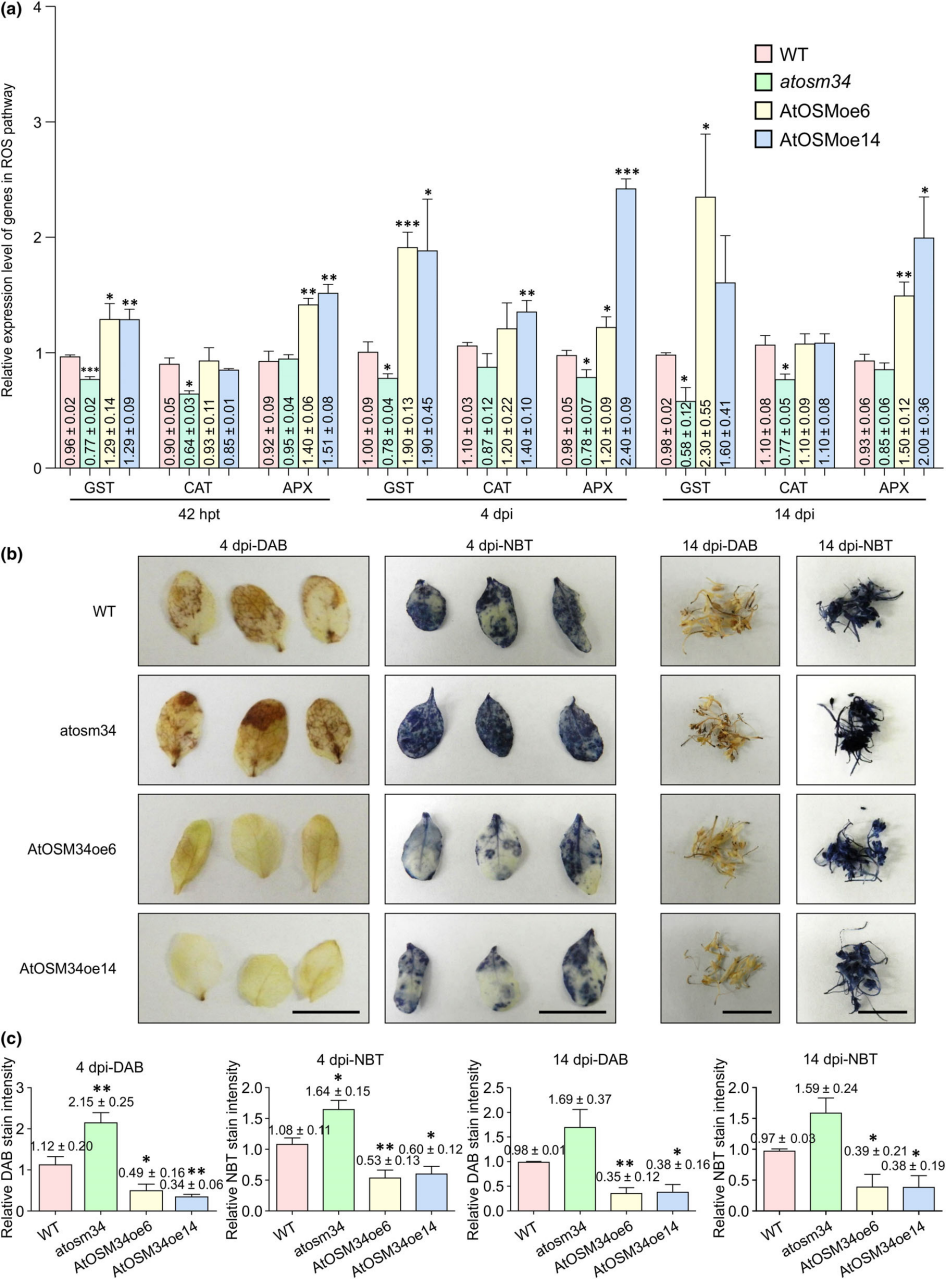
AtOSM34 inhibits ROS accumulation. (a) Relative expression level of genes in the ROS pathway in WT, *atosm34* and AtOSM34 transgenic overexpression Arabidopsis plants at 42 h posttransfection (hpt), 4 and 14 d postinfiltration (dpi). Total RNA was extracted from the protoplast transfected by TuMV‐GFP at 42 hpt, leaves inoculated with TuMV‐GFP at 4 dpi or inflorescence tip tissues of plants inoculated with TuMV‐GFP at 14 dpi. Reverse transcription quantitative polymerase chain reaction was performed with specific primers indicated in Supporting Information Table S1. (b) Detection of hydrogen peroxide by DAB staining and detection of superoxide anion by NBT staining in Col‐0, *atosm34* and transgenic plants overexpressing AtOSM34 at 4 dpi (leaves) and 14 dpi (stem tops). Scale bar, 1 cm. (c) The relative DAB and NBT staining intensity of Col‐0 (WT), *atosm34* and transgenic plants overexpressing AtOSM34 at 4 and 14 dpi. The relative DAB and NBT staining intensity was quantified by ImageJ software. In (a, c), values are presented as arbitrary units relative to WT. Statistically significant differences, determined by unpaired two‐tailed Student's *t*‐test comparing WT and transgenic plants are indicated: *, *P* < 0.05; **, *P* < 0.01; ***, *P* < 0.001.

OSM and OLPs are known to confer plant tolerance against biotic and abiotic stresses through the integrated activation of multiple components of the defense signaling cascade (Hakim *et al*., 2018). Overexpression of OSM or OLPs in diverse plants results in an increase in ROS‐scavenging enzymes and reduction in ROS production under stress (Chowdhury *et al*., 2017; Bashir *et al*., 2020). It is possible that AtOSM34 may also modulate the ROS pathway in favor of TuMV infection. To test this idea, we analyzed the expression of three marker genes for ROS during infection by TuMV, including CAT, GST and APX which encode catalase, glutathione *S*‐transferase and ascorbate peroxidase, respectively. These three enzymes are important components of the ROS‐scavenging system (Mittler, 2017; Wahibah *et al*., 2018). Transcription levels of these genes were significantly downregulated in the *atosm34* mutants, compared to those in WT Arabidopsis plants (Fig. 10a). By contrast, the expression levels of these genes were significantly upregulated in AtOSM34 overexpression lines (Fig. 10a). We further assessed the accumulation level of hydrogen peroxide and superoxide by DAB and NBT staining. Compared with the WT plants, significantly higher levels of hydrogen peroxide and superoxide were detected in the *atosm34* mutants at 4 dpi and 14 dpi, while significantly lower levels of ROS accumulation were evident in the transgenic lines overexpressing AtOSM34 (Fig. 10b,c). Taken together, these results support that AtOSM34 may also indirectly promote TuMV infection by reducing ROS‐mediated antiviral resistance.

## Discussion

In this study, we investigated the possible involvement of AtOSM34 and AtOLP in TuMV infection. These two PR‐5/TLP family proteins are Arabidopsis orthologs of three significantly differentially accumulated proteins we identified previously in the PD‐enriched fraction of *N. benthamiana* plants after TuMV infection (Park *et al*., 2017). We present evidence that knockdown of *AtOSM34* restricts, while its overexpression promotes, TuMV infection in Arabidopsis (Figs 2, 4) and that neither downregulation nor upregulation of *AtOLP* affects TuMV infection (Fig. S5d–i). These data suggest that TuMV selectively subverts AtOSM34 to assist its infection. Given that OSM and related TLPs are PR‐5/TLP family proteins, and PR proteins are considered as part of the plant defense system against various biotic and abiotic stresses, the finding from this study that AtOSM34 acts as a proviral factor in TuMV infection was not what we expected.

AtOSM34 was first discovered by screening of a phage library of Arabidopsis cDNAs with oligo DNAs designed from highly conserved regions in tobacco OSM and OLPs and named after one of the selected clones (Capelli *et al*., 1997). The *A. thaliana* genome encodes a total of 22 PR‐5/TLP family proteins but only one OSM (AtOSM34) (Capelli *et al*., 1997; Faillace *et al*., 2019). This single copy gene expresses a putative proprotein of 244 aa with a calculated molecular mass of 26.5 kDa including the predicted N‐terminal signal peptide (22 aa) and C‐terminal propeptide sequence (19 aa) (Capelli *et al*., 1997). The only characterized function of AtOSM34 is its positive regulation of abscisic acid (ABA) mediated stress responses, likely through the regulation of ABA‐induced proline synthesis (Park & Kim, 2021). PR‐5/TLP superfamily proteins including OLPs, TLPs and ZLPs are induced in response to abiotic and biotic stresses including viral infection (Sinha *et al*., 2014; Kumar *et al*., 2015; Hakim *et al*., 2018; Bashir *et al*., 2020). For instance, tobacco mosaic virus (TMV) infection induces the expression of a PR‐5/TLP protein in tobacco plants (Cornelissen *et al*., 1986; Stintzi *et al*., 1991; Albrecht *et al*., 1992). Infection by cucumber mosaic virus (CMV) boosts *AtTLP1* expression in *Nicotiana tabacum* and this TLP specifically interacts with the CMV movement protein and CP (Kim *et al*., 2005). Together with several other *PR* genes, *PR‐5* is significantly upregulated in pepper and tomato plants in response to infections by pepper mild mottle virus and tomato spotted wilt virus (TSWV) (Elvira *et al*., 2008; Padmanabhan *et al*., 2019). However, in some other plant‐virus combinations, some *TLP* genes may be downregulated. For instance, Aseel *et al*. (2023) recently reported that TMV infection reduces *PR‐5* expression in tomato at an early infection stage (before 9 dpi), while CMV infection stimulates *PR‐5* transcription (before 4 dpi). In this study, we demonstrate that TuMV infection upregulates *AtOSM34* (Fig. 1b) but downregulates *NtOLP* (Fig. S2b). Apparently, differential expression of *PR‐5/TLP* family genes in response to virus infection is probably dependent on specific genes and viruses.

Transgenic plants overexpressing PR‐5/TLPs often show enhanced tolerance to various biotic and abiotic stresses by reducing ROS production, limiting lipid peroxidation, increasing proline content and ROS‐scavenging enzyme activity, regulating phytohormone (ABA, ethylene and auxin)‐mediated signaling pathways, and upregulating the expression of defense‐related genes (Mahdavi *et al*., 2012; Acharya *et al*., 2013; Chowdhury *et al*., 2017; Le *et al*., 2018; He *et al*., 2021; Zhu *et al*., 2021; Li *et al*., 2023). One of the most characterized activities of PR‐5/TLP family proteins is their broad‐spectrum antifungal activity. Although the exact molecular mechanisms against fungi remain unclear, it has been suggested that OSMs and related TLPs interact with the plasma membrane of fungi to disrupt the plasma membrane and cell wall interface, subsequently leading to leakage of fungal cells and restriction of hyphal growth and spore germination (Kumar *et al*., 2015; Hakim *et al*., 2018). There is not much direct evidence available to support that PR‐5/TLP family proteins have antiviral activity. One piece of favorable evidence is that transgenic tomato plants overexpressing PR‐5 are more tolerant to TSWV infection (Padmanabhan *et al*., 2019). Nevertheless, transgenic overexpression of an OSM in barley does not enhance resistance or tolerance to barley yellow dwarf virus and wheat dwarf virus (Viktorova *et al*., 2019). In this study, transgenic overexpression of AtOLP in Arabidopsis does not reduce TuMV infection (Fig. S5d–i) and overexpression of AtOSM34 even promotes TuMV infection (Fig. 4). More studies are needed to elucidate the role of PR‐5/TLP family proteins in plant‐virus infection.

To better understand the mechanism by which AtOSM34 promotes TuMV infection, we conducted a protoplast transfection assay with Arabidopsis mesophyll protoplasts. As this system offers a homogeneous cell population with high percentage of synchronous infection and effects free from other cells and tissues, it has been established as an excellent tool for characterization of early molecular events, such as virus replication, on potyvirus infection (Dai & Wang, 2022a). We found that knockdown of *AtOSM34* in Arabidopsis inhibits viral RNA accumulation by *c*. 35% in protoplasts at 42 hpt (Fig. 2d), while overexpression of AtOSM34 in *N. benthamiana* enhances viral RNA accumulation by *c*. ninefold at 68 hpi (Figs 5c, S10b). We speculated that AtOSM34 may promote TuMV replication. To address this possibility, we investigated the subcellular distribution of AtOSM34 in TuMV‐infected cells. Interestingly, AtOSM34‐YFP highlights large aggregated structures in the cytoplasm that colocalize with 6K2‐mCherry‐containing vesicles (Fig. 9a). As briefly mentioned above, 6K2‐labelled vesicle‐like structures are the hallmark of potyviral VRCs that are ER‐derived and often target chloroplasts (Wei & Wang, 2008; Cotton *et al*., 2009; Wei *et al*., 2010a). To identify possible viral interactors of AtOSM34, protein–protein interaction assays (mYTH, BiFC, and Co‐IP) were conducted. We found that AtOSM34 interacts with TuMV protein 6K2 via its domains DII and DIII (Fig. 8) and the AtOSM34 and 6K2 interaction complexes are indeed colocalized with 6K2‐induced chloroplast‐associated vesicles in the presence of TuMV infection (Fig. 9b). These data support the idea that AtOSM34 may be recruited to the chloroplast‐associated VRCs via its interaction with 6K2 to facilitate TuMV replication.

Chloroplasts are the major site for ROS production, which is regulated by a diversified ROS‐scavenging system (Mittler, 2017). In general, ROS appear to play an inhibitory role in viral infection (Li *et al*., 2016a). A recent study showed that silencing of *NbLHCB3*, which encodes the light‐harvesting Chl*a*/*b* complex protein 3 of photosystem II, enhances ROS accumulation and inhibits TuMV infection in *N. benthamiana* (Qiu *et al*., 2021). Chemical treatment to inhibit ROS or silencing of genes responsible for ROS production compromises TuMV resistance induced by silencing of *NbLHCB3* (Qiu *et al*., 2021). During the coevolutionary arms race, viruses have likely evolved mechanisms to antagonize the antiviral role of ROS. It has been reported that red clover necrotic mosaic virus and brome mosaic virus could harness ROS to enhance viral replication (Hyodo *et al*., 2017a,b). On the other hand, ROS may be conducive to aphid‐borne viral spread (Berthelot *et al*., 2019; Guo *et al*., 2019). An increasing body of evidence points to a link between OSMs and the ROS pathway. OSMs may prevent the accumulation of hydrogen peroxide by increasing antioxidant scavenger enzymes (Wan *et al*., 2017). In agreement with this, overexpression of a tobacco OSM in transgenic plants protects them from different stresses by increasing scavenging enzyme activity and reducing ROS production (Hakim *et al*., 2018; Bashir *et al*., 2020). In this study, we found that knockdown of *AtOSM34* downregulates all three ROS‐scavenging pathway genes examined, and significantly increases ROS accumulation (Fig. 10), whereas overexpression of *AtOSM34* upregulates these genes and reduces ROS accumulation (Fig. 10). Thus, it is reasonable to suggest that the recruitment of AtOSM34 by 6K2 to the chloroplast‐associated VRC may contribute to inhibition of ROS accumulation and subsequently mitigation of ROS‐mediated antiviral response.

In this study, we confirm that AtOSM34 and AtOLP from Arabidopsis and their orthologs from *N. benthamiana* are all PD‐located proteins (Figs 1a, S2a, S3a–c). Since all plant viruses must pass through PD to enter neighboring cells for systemic infection, we examined if these OLPs are involved in TuMV intercellular movement. Transient expression of these OLPs indeed promotes TuMV cell‐to‐cell movement (Figs 5a, S6a). Callose, a linear β‐1,3‐glucan, surrounding the PD neck region, is considered an important component to control the PD size exclusion limit (S. W. Wu *et al*., 2018). The turnover of callose at PD modulates the intercellular trafficking of molecules through PD. The degradation of callose is mediated by β‐1,3‐glucanases (BGs) (Zavaliev *et al*., 2011; Amsbury *et al*., 2017; S. W. Wu *et al*., 2018). Previous studies have shown that some TLPs can bind to β‐1,3‐glucan and exhibit BG activity (Grenier *et al*., 1999; Osmond *et al*., 2001; Menu‐Bouaouiche *et al*., 2003). A recent study showed that overexpression of two TLPs in tomato plants enhanced BG activity and conferred resistance to five soil‐borne diseases (three fungal and two bacterial pathogens) (Li *et al*., 2023). This prompted us to check PD callose and permeability in leaf tissues of transgenic AtOSM34 and AtOLP‐overexpression plants. Indeed, overexpression of AtOSM34 decrease deposition of callose at PD and increases PD permeability (Figs 6b–g, 7). These data suggest that AtOSM34 and AtOLP may function directly as BG or indirectly by recruitment of BGs to degrade PD callose, thereby increasing PD permeability to facilitate viral intercellular movement.

In summary, we show that AtOSM34 is a proviral host factor for TuMV infection. Upon TuMV infection, *AtOSM34* is selectively upregulated, leading to AtOM34 accumulation. As a PD‐localized protein, AtOSM34 may regulate PD permeability to facilitate TuMV intercellular movement. On the other hand, AtOSM34 is also recruited to the VRC via the interaction with 6K2 to promote viral replication. Moreover, upregulation of *AtOSM34* further activates the expression of genes encoding ROS‐scavenging enzymes to reduce ROS accumulation, which may represent a counteracting mechanism to inhibit ROS‐mediated antiviral response.

## Competing interests

None declared.

## Author contributions

AW and RH designed the project; RH carried out experiments with assistance from YL; AW and MAB supervised the work; all authors analyzed and discussed the data; RH and AW wrote the manuscript. All the authors reviewed, revised and approved the manuscript.

## Supporting information


**Fig. S1** Amino acid sequence alignment and phylogenetic tree of AtOSM34, AtOLP, NbOSM, NbOLP1 and NbOLP2.
**Fig. S2** AtOLP is a PD‐located protein and *AtOLP* is downregulated by TuMV infection.
**Fig. S3** NbOSM, NbOLP1 and NbOLP2 are PD‐located proteins, and their expression is differentially regulated in response to TuMV infection.
**Fig. S4** Identification of the *atosm34* mutant and generation of transgenic Arabidopsis lines overexpressing AtOSM34.
**Fig. S5** Identification of *atolp* mutants, generation of transgenic Arabidopsis lines overexpressing AtOLP and TuMV infection assays on *atolp* mutants and AtOLP overexpresison Arabidopsis lines.
**Fig. S6** Overexpression of OLPs facilitates TuMV intercellular movement.
**Fig. S7** Overexpression of AtOLP reduces PD callose deposition and increases PD permeability.
**Fig. S8** Detection of the interaction of AtOMS34 or AtOLP with TuMV proteins.
**Fig. S9** Detection of the interactions of AtOSM34 domains with TuMV 6K2 and VPg.
**Fig. S10** Transient expression of the AtOSM34 DII deletion mutant on TuMV intercellular movement and viral accumulation.
**Table S1** List of primers used in this study.
Please note: Wiley is not responsible for the content or functionality of any Supporting Information supplied by the authors. Any queries (other than missing material) should be directed to the *New Phytologist* Central Office.

## Data Availability

The data that support the findings of this study are available on request from the corresponding author (Figs S1–S10; Table S1).
