# Supplementary material for: Turnip mosaic virus selectively subverts a PR‐5 thaumatin‐like, plasmodesmal protein to promote viral infection
Source: New Phytol. 2024 Nov 12;245(1):299–317. doi: 10.1111/nph.20233 (PMC11617660; doi:10.1111/nph.20233)
Supplement: Supplementary file 1 — Fig. S1 Amino acid sequence alignment and phylogenetic tree of AtOSM34, AtOLP, NbOSM, NbOLP1 and NbOLP2. Fig. S2 AtOLP is a PD‐located protein and AtOLP is downregulated by TuMV infection. Fig. S3 NbOSM, NbOLP1 and NbOLP2 are PD‐located proteins, and their expression is differentially regulated in response to TuMV infection. Fig. S4 Identification of the atosm34 mutant and generation of transgenic Arabidopsis lines overexpressing AtOSM34. Fig. S5 Identification of atolp mutants, generation of transgenic Arabidopsis lines overexpressing AtOLP and TuMV infection assays on atolp mutants and AtOLP overexpresison Arabidopsis lines. Fig. S6 Overexpression of OLPs facilitates TuMV intercellular movement. Fig. S7 Overexpression of AtOLP reduces PD callose deposition and increases PD permeability. Fig. S8 Detection of the interaction of AtOMS34 or AtOLP with TuMV proteins. Fig. S9 Detection of the interactions of AtOSM34 domains with TuMV 6K2 and VPg. Fig. S10 Transient expression of the AtOSM34 DII deletion mutant on TuMV intercellular movement and viral accumulation. Table S1 List of primers used in this study. Please note: Wiley is not responsible for the content or functionality of any Supporting Information supplied by the authors. Any queries (other than missing material) should be directed to the New Phytologist Central Office. [file NPH-245-299-s001.docx]

## *New Phytologist* Supporting Information

Article title: Turnip mosaic virus selectively subverts a PR-5 thaumatin-like, plasmodesmal protein to promote viral infection

Authors: Rongrong He, Yinzi Li, Mark A. Bernards, Aiming Wang

Article acceptance date: 27 September 2024

The following Supporting Information is available for this article:

**Fig. S1** Amino acid sequence alignment and phylogenetic tree of AtOSM34, AtOLP, NbOSM, NbOLP1 and NbOLP2.

**Fig. S2** AtOLP is a PD-located protein and AtOLP is downregulated by TuMV infection.

**Fig. S3** NbOSM, NbOLP1 and NbOLP2 are PD-located proteins, and their expression is differentially regulated in response to TuMV infection.

**Fig. S4** Identification of the *atosm34* mutant and generation of transgenic Arabidopsis lines overexpressing AtOSM34.

**Fig. S5** Identification of *atolp* mutants, generation of transgenic Arabidopsis lines overexpressing AtOLP and TuMV infection assays on *atolp* mutants and AtOLP overexpresison Arabidopsis lines.

**Fig. S6** Overexpression of OLPs facilitates TuMV intercellular movement.

**Fig. S7** Overexpression of AtOLP reduces PD callose deposition and increases PD permeability.

**Fig. S8** Detection of the interaction of AtOMS34 or AtOLP with TuMV proteins.

**Fig. S9** Detection of the interactions of AtOSM34 domains with TuMV 6K2 and VPg.

**Fig. S10** Transient expression of the AtOSM34 DII deletion mutant on TuMV intercellular movement and viral accumulation.

**Table S1** List of primers used in this study.

**Fig. S1** Amino acid sequence alignment and phylogenetic tree of AtOSM34, AtOLP, NbOSM, NbOLP1 and NbOLP2. (a) Amino acid identity (%) shared among AtOSM34, AtOLP, NbOSM, NbOLP1 and NbOLP2 created using Clustal 2.1 software ([www.clustal.org/clustal2/](http://www.clustal.org/clustal2/)). (b) Multiple sequence alignment of amino acid sequences of AtOSM34, AtOLP, NbOSM, NbOLP1 and NbOLP2 using the CLUSTALW online ([www.genome.jp/tools-bin/clustalw](http://www.genome.jp/tools-bin/clustalw)). (c) A Phylogenetic tree of AtOSM34, AtOLP, NbOSM, NbOLP1 and NbOLP2 created using MEGA11 ([www.megasoftware.net](http://www.megasoftware.net)).

**
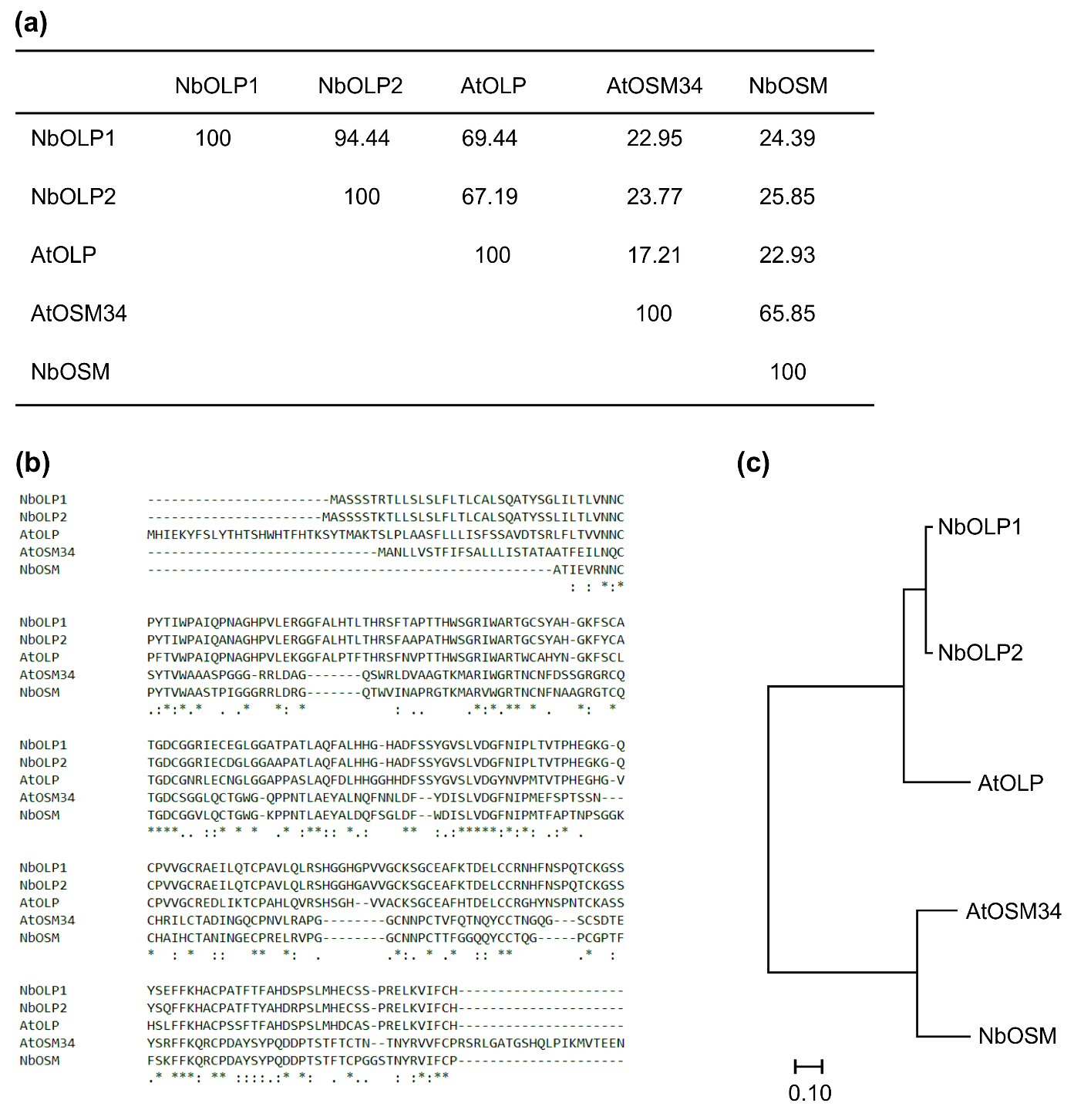
**

**Fig. S2** AtOLP is a PD-located protein and AtOLP is downregulated by TuMV infection. (a) Colocalization of AtOLP-YFP with callose binding dye aniline blue (upper panel) and the PD marker AtPDLP5-CFP (lower panel). AtOLP-YFP was transiently expressed in *N. benthamiana* leaves, and then infiltrated with aniline blue at 48 hpi and incubated for 5 min before imaging (upper panel). Transient co-expression of AtOLP-YFP and AtPDLP5-CFP in *N. benthamiana* leaf cells (lower panel). White arrows indicate the representative PD localizations. Images were taken at 48 hpi. DIC, differential interference contrast. Scale bar, 10 µm. (b) Relative mRNA expression level of *AtOLP* in the Arabidopsis inoculated leaves (at 4 dpi) and stalk tip tissues (at 7 dpi and 14 dpi) after TuMV inoculation compared with that in corresponding tissues from mock-inoculated control. Data are means of results from three independent experiments, with SE (*n* = 3). Statistically significant difference, determined by unpaired two-tailed Student's *t*-test, is indicated: ***, *P* < 0.001; NS, not significant.

**
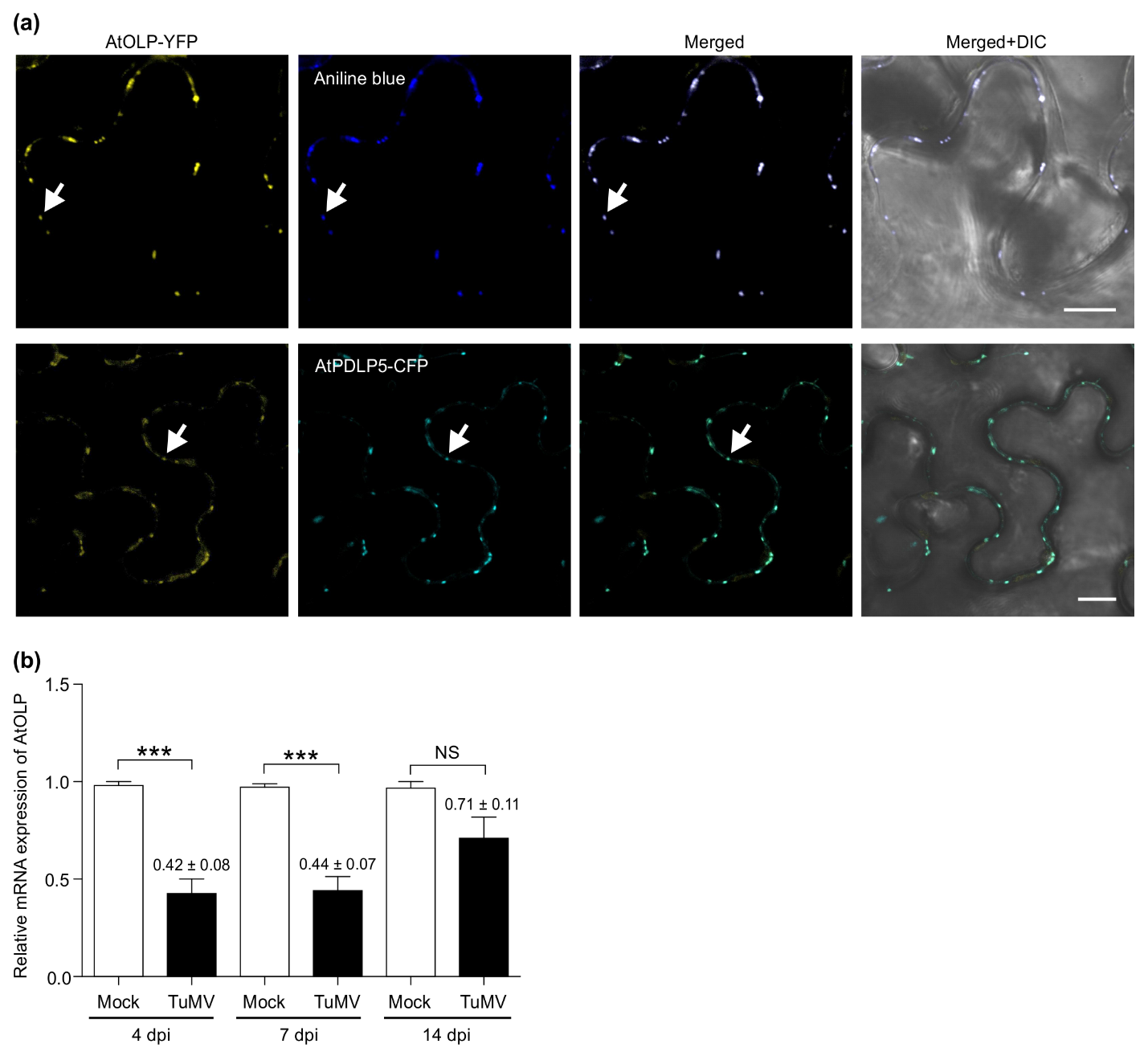
**

**Fig. S3** NbOSM, NbOLP1 and NbOLP2 are PD-located proteins, and their expression is differentially regulated in response to TuMV infection. (a) Colocalization of NbOSM-YFP with callose binding dye aniline blue (upper panel) and the PD marker AtPDLP5-CFP (lower panel). (b) Colocalization of NbOLP1-YFP with callose binding dye aniline blue (upper panel) and the PD marker AtPDLP5-CFP (lower panel). (c) Colocalization of NbOLP2-YFP with callose binding dye aniline blue (upper panel) and the PD marker AtPDLP5-CFP (lower panel). (d,e) Relative mRNA expression levels of *NbOSM* (d) and *NbOLP1/2* (e) in the inoculated leaves at 4 dpi and systemically infected leaves at 7 and 14 dpi from *N. benthamiana* plants inoculated with TuMV-GFP, compared with that in corresponding tissues from mock-inoculated control plants. Values are means ± SE (*n* = 3) and are presented as arbitrary units relative to mock. The experiment was repeated three times and each consisted of three biological replicates with each including a pooled sample from three plants. *NbActin* transcripts in the same sample were used as an internal control. Statistically significant differences, determined by unpaired two-tailed Student's *t*-test comparing mock and TuMV-inoculated plants are indicated: *, *P* < 0.05; **, *P* < 0.01. In (a,b,c), *N. benthamiana* leaves were agroinfiltrated alone with NbOSM-YFP, NbOLP1-YFP, or NbOLP2-YFP for transient expression, or together with AtPDLP for transient co-expression. The leaf tissues transiently expressing NbOSM-YFP, NbOLP1 or NbOLP2 were further infiltrated with aniline blue at 48 hpi and incubated for 5 min before imaging. All images were taken at 48 hpi. DIC, differential interference contrast. Scale bar, 10 µm.

**
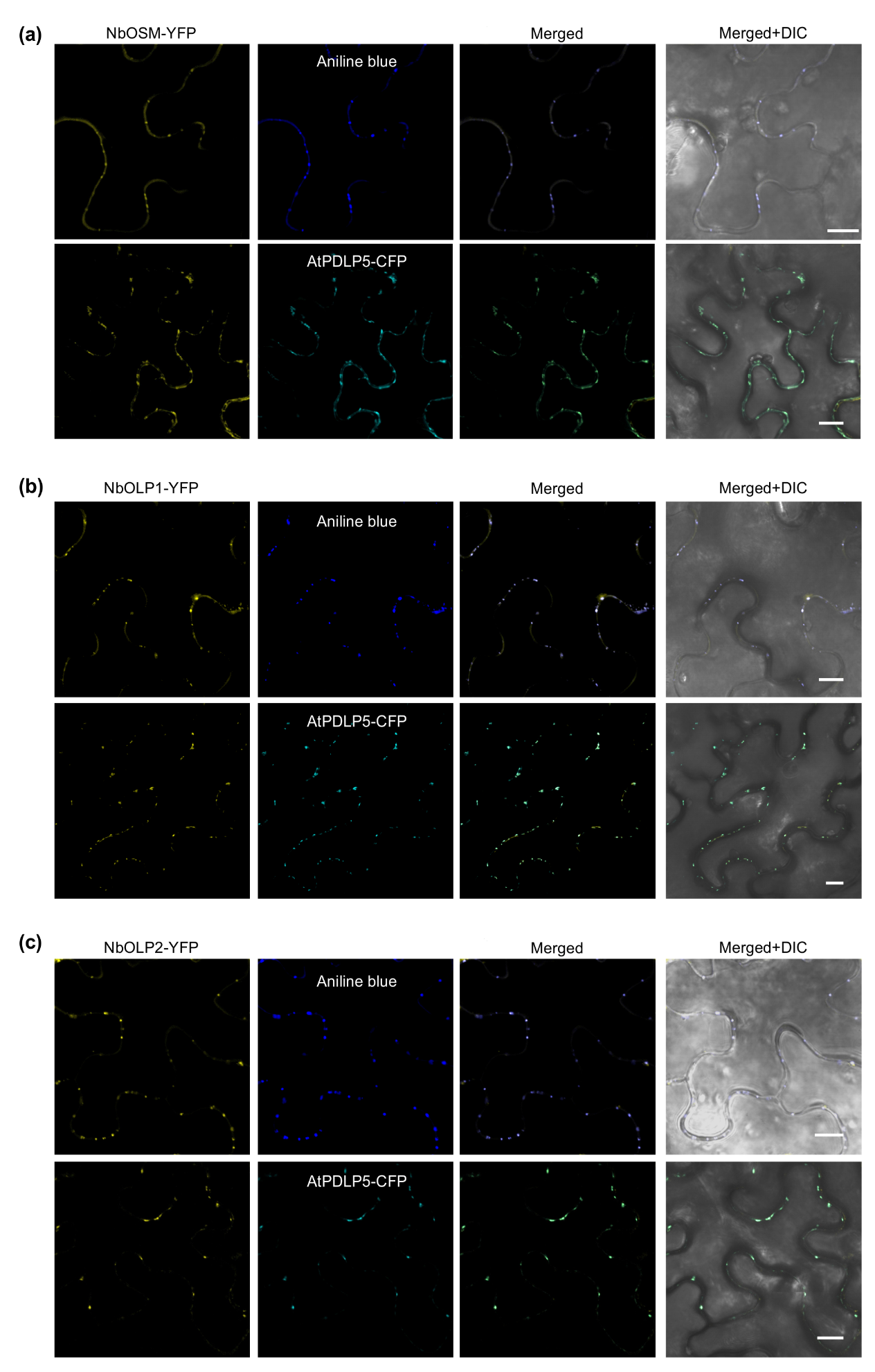
**

**

**

**Fig. S4** Identification of the *atosm34* mutant and generation of transgenic Arabidopsis lines overexpressing AtOSM34. The relative position of T-DNA insertion site in the T-DNA line CS916967 is shown. (b) PCR genotyping of the *atosm34* mutant. (c) RT-PCR detection of *AtOSM34* transcripts in WT and *atosm34* Arabidopsis. (d) RT-qPCR analysis of *AtOSM34* transcripts in WT and *atosm34* plants. RT-qPCR was performed using cDNA derived from leaf samples with specific primers. *AtActinII* was used as an internal control. Values are means with SE (*n* = 3) and are presented as arbitrary units relative to WT. Statistically significant differences, determined by unpaired two-tailed Student's t-test, are indicated: **, *P* < 0.01. (e) Phenotypes of 14-day old WT and *atosm34* plants. (f) RT-qPCR analysis of mRNA expression of *AtOSM34* in WT, and two representative transgenic Arabidopsis plants overexpressing AtOSM34. RT-qPCR was performed using cDNA derived from leaf samples with specific primers. *AtActinII* was used as an internal control. Values are means with SE (*n* = 3) and are presented as arbitrary units relative to WT. This experiment was repeated three time. Statistically significant differences, determined by unpaired two-tailed Student's t-test, are indicated: ***, *P* < 0.001. (g) Phenotypes of 14-day old WT and transgenic plants overexpressing AtOSM34 under normal growth conditions.

**
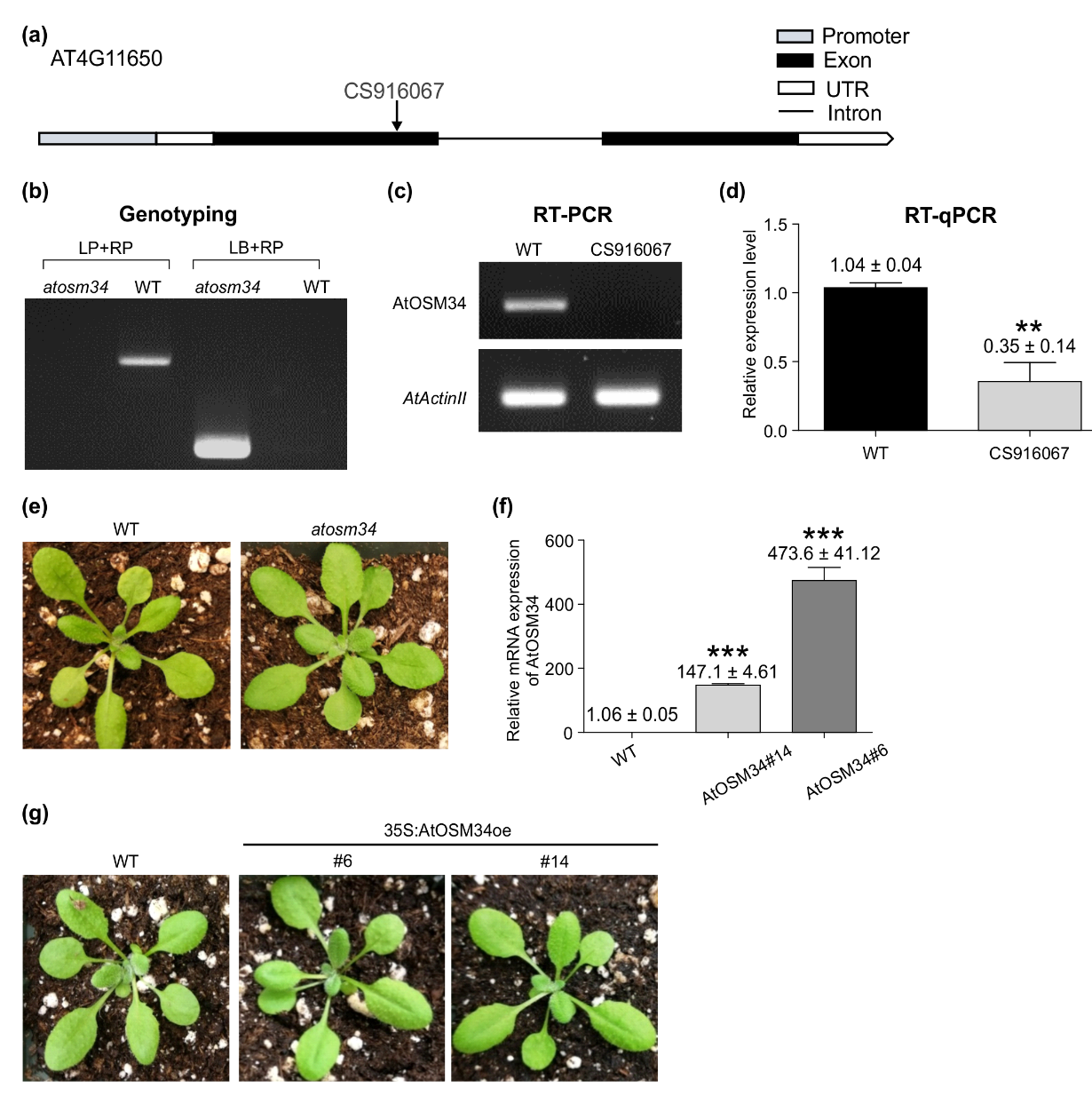
**

**Fig. S5** Identification of *atolp* mutants, generation of transgenic Arabidopsis lines overexpressing AtOLP and TuMV infection assays on *atolp* mutants and AtOLP overexpresison Arabidopsis lines. The relative position of T-DNA insertion site in two T-DNA insertion lines (CS850431 and CS819509) is indicated. (b) RT-qPCR analysis of *AtOLP* transcripts in WT Col-0 and two *atolp* mutant lines (left), and WT Col-0 and two representative transgenic lines overexpressing AtOLP (right). RT-qPCR was performed using cDNA derived from leaf samples with specific primers. *AtActinII* was used as an internal control. Values are means with SE (*n* = 3) and are presented as arbitrary units relative to WT. This experiment was performed three time. Statistically significant differences, determined by unpaired two-tailed Student's *t*-test, are indicated: ***, *P* < 0.001. (c) Phenotypes of 14-day old WT, *atolp* mutant, AtOLP overexpression plants. (d) Phenotypes of WT and *atolp* plants infected by TuMV. (e) RT-qPCR analysis of TuMV RNA levels in WT and *atolp* plants. Viral RNA was quantified by RT-qPCR with primers specific for the *CP* coding region. *AtActinII* was used as an internal control. Values are means with SE (*n* = 3) and are presented as arbitrary units relative to WT. No statistically significant differences found by unpaired two-tailed Student's *t*-test. (f) Phenotypes of WT and AtOLP overexpression plants infected by TuMV. (g) RT-qPCR analysis of TuMV RNA levels in WT and two AtOLP overexression lines. Viral RNA was quantified by RT-qPCR with primers specific for the *CP* coding region. *AtActinII* was used as an internal control. Values are means with SE (*n* = 3) and are presented as arbitrary units relative to WT. No statistically significant differences found by unpaired two-tailed Student's *t*-test. (h) TuMV transfection assay with protoplasts isolated from WT and *atolp* mutant plants. Total RNA was isolated from protoplasts transfected by TuMV-GFP at 42 hpt. Viral RNA was quantified by RT-qPCR with primers specific for the *CP* coding region. Data were normalized against *AtActinII* as the internal reference. Values are means with SE (*n* = 3) and are presented as arbitrary units relative to WT. No statistically significant differences found by unpaired two-tailed Student's *t*-test.

**
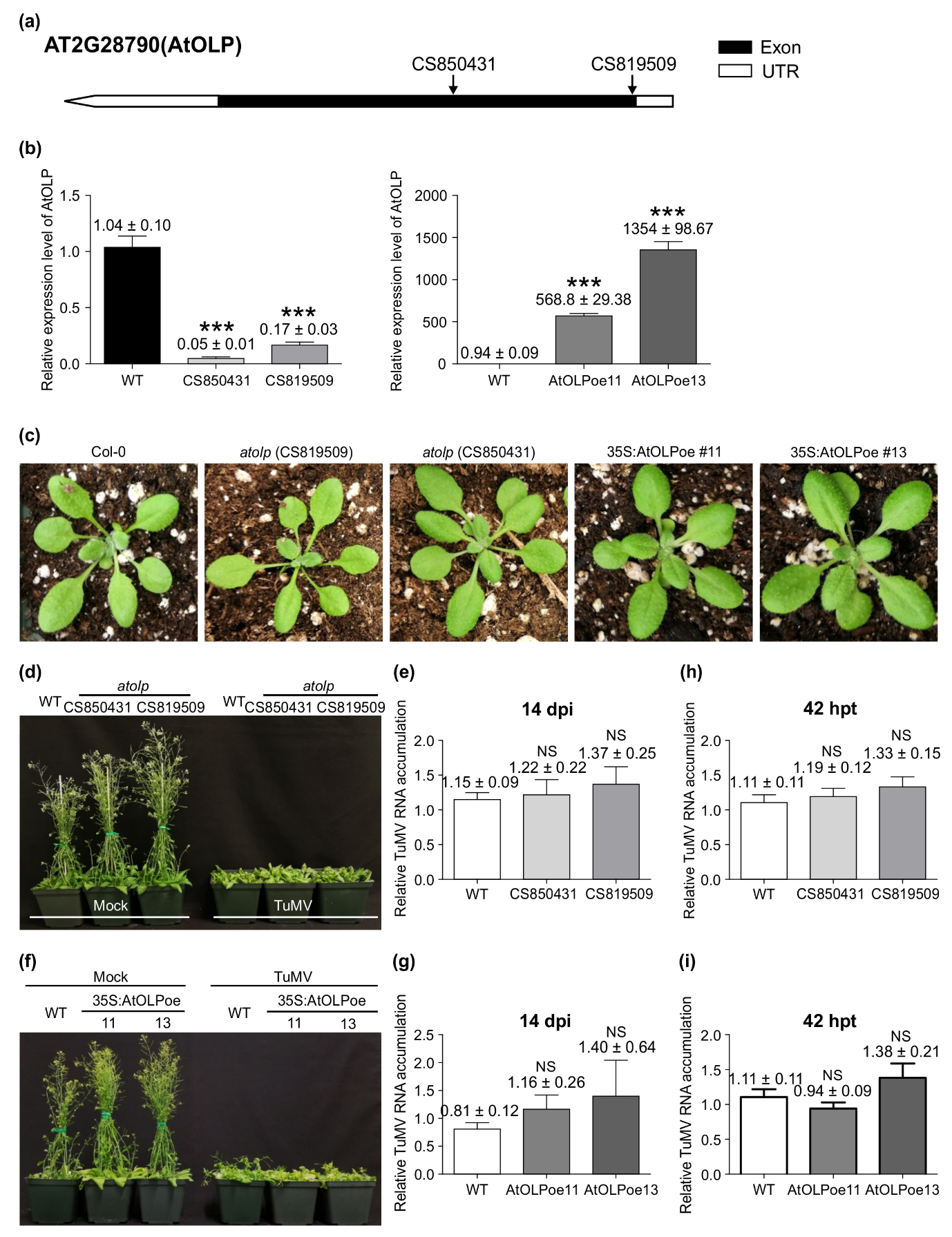
**

**Fig. S6** Overexpression of OLPs facilitates TuMV intercellular movement. (a) Confocal microscopy observation of viral intercellular movement *in N. benthamiana* leaf tissues. Agrobacterial cells harboring the plasmid TuMV:: GFP//mCherry-HDEL (OD_600_ =0.001) were coinfiltrated into leaves of *N. benthamiana* with either empty vector (EV) (upper panels), AtOLP-Flag-4×Myc (middle panels), NbOLP1-Flag-4×Myc (lower panels), or NbOLP2-Flag-4×Myc (bottom panels). Red fluorescence emitted by mCherry from the mCherry-HDEL expression cassette, and green fluorescence by GFP from the recombinant TuM-GFP genome. Cells emitting both red and green fluorescence represent primarily infected ones whereas those emitting GFP only are secondarily infected cells. Images were taken at 66 hpi. Scale bar, 100 µm. (b) Immunoblotting analysis of viral GFP protein accumulation in the infiltrated leaf tissues from *N. benthamiana* plants at 68 hpi. Total proteins were extracted from the infiltrated leaf tissues at 68 hpi, and immunoblotted with anti-GFP and anti-Myc antibodies, respectively. The blots were quantified by ImageJ software (mean ± SD; *n* = 3). The Coomassie Brilliant Blue R-250-stained Rubisco large subunit (CBB) serves as a loading control.

**
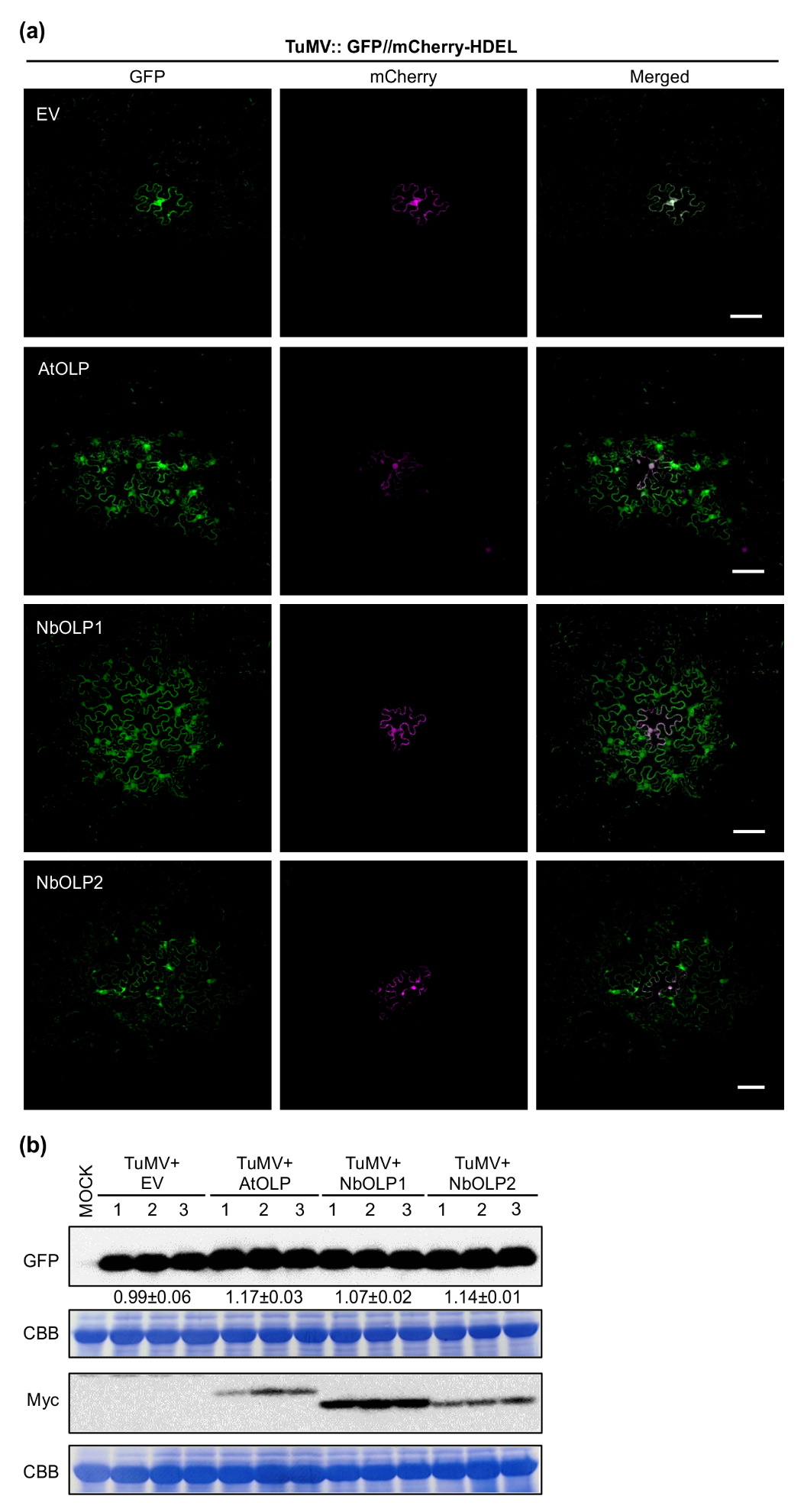
**

**Fig. S7** Overexpression of AtOLP reduces PD callose deposition and increases PD permeability. (a,c,d) Comparison of callose deposition level in WT and transgenic AtOLP overexpression Arabidopsis leaf tissues. Two transgenic lines, AtOLPoe11 and AtOLPoe13 were used in this experiment. (a) Confocal images of callose staining. Scale bar, 20 µm. (c) Quantification of fold change of callose deposition number. (d) Quantification analysis of the callose deposition intensity. (b,e,f) Comparison of PD permeability of WT and transgenic AtOLP overexpression Arabidopsis leaves by CFDA-based DANS dye loading assay. (b) Confocal images of CFDA movement. Scale bar, 200 µm. (e,f) Quantification analysis of diffusion area (e) and fluorescence intensity of CFDA(f). In (c,d,e,f), values are means with SE from three independent experiments. In each experiment, 10 plants were used for each treatment. Statistically significant differences, determined by unpaired two-tailed Student's *t*-test comparing WT and overexpression plants are indicated: *, *P* < 0.05; **, *P* < 0.01; ***, *P* < 0.001.

**
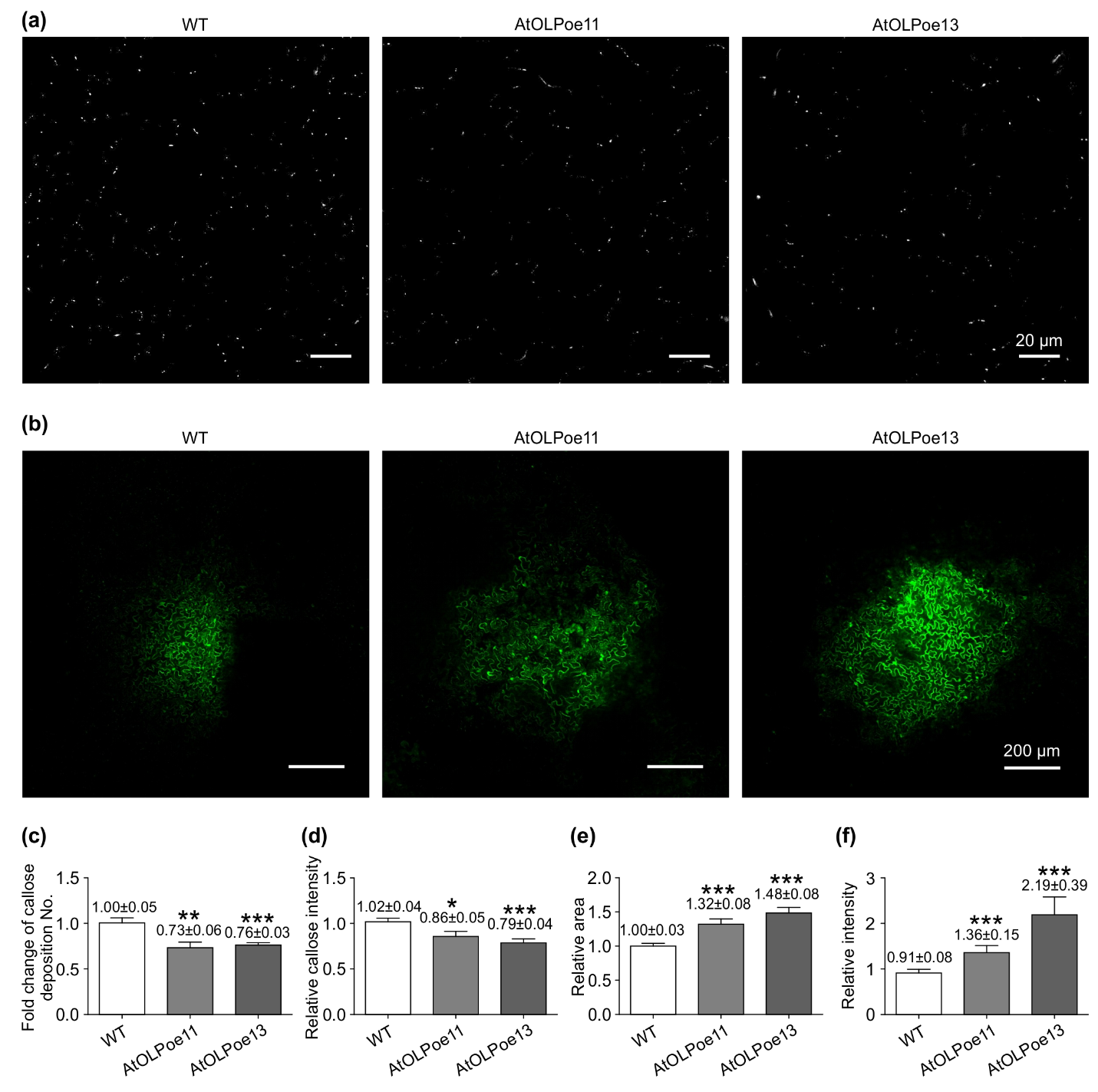
**

**Fig. S8** Detection of the interaction of AtOMS34 or AtOLP with TuMV proteins. (a) BiFC assay. No interactions were found between YN-AtOSM34 and PIPO-YC, or between YN-AtOLP and VPg-YC or 6K2-YC. (b) Confirmation of expression of YN-AtOLP and 6K2-YC in the BiFC assay. (c) Co-IP assay of interactions between AtOSM34 and VPg. No positive interaction was detected.

**
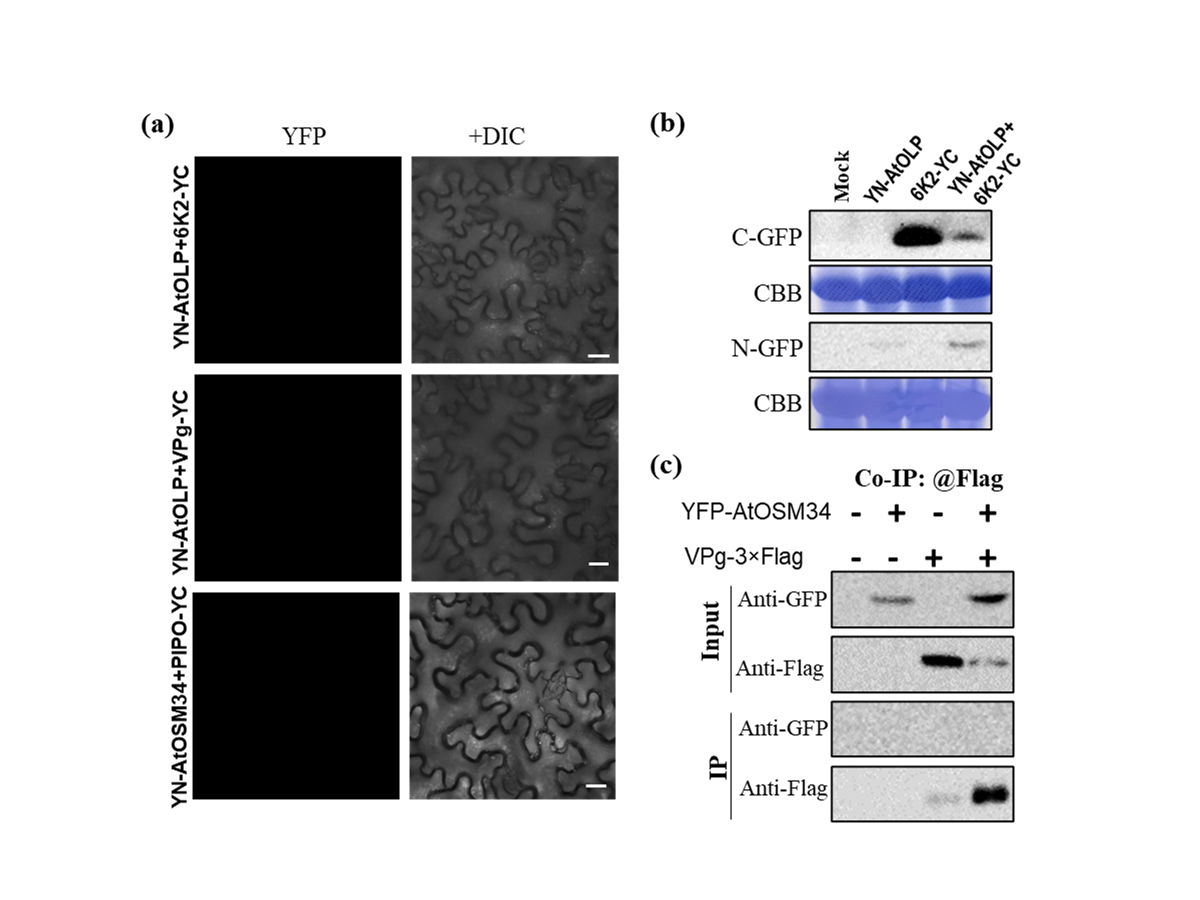
**

**Fig. S9** Detection of the interactions of AtOSM34 domains with TuMV 6K2 and VPg. (a) BiFC assay in *N*. *benthamiana* leaf cells. No interactions were detected between OSM domains SP, DI, or DIII with 6K2 or VPg. Scale bar, 20 µm. (b,c,d) mYTH assay of the interactions of DI (b) and SP (c) of AtOSM34, empty vector (d) of pPR3-N with TuMV 6K2 and VPg. No positive interactions were detected. (e) Co-IP assay of interactions between AtOSM34-DII, DIII and VPg. No positive interaction were detected.

**
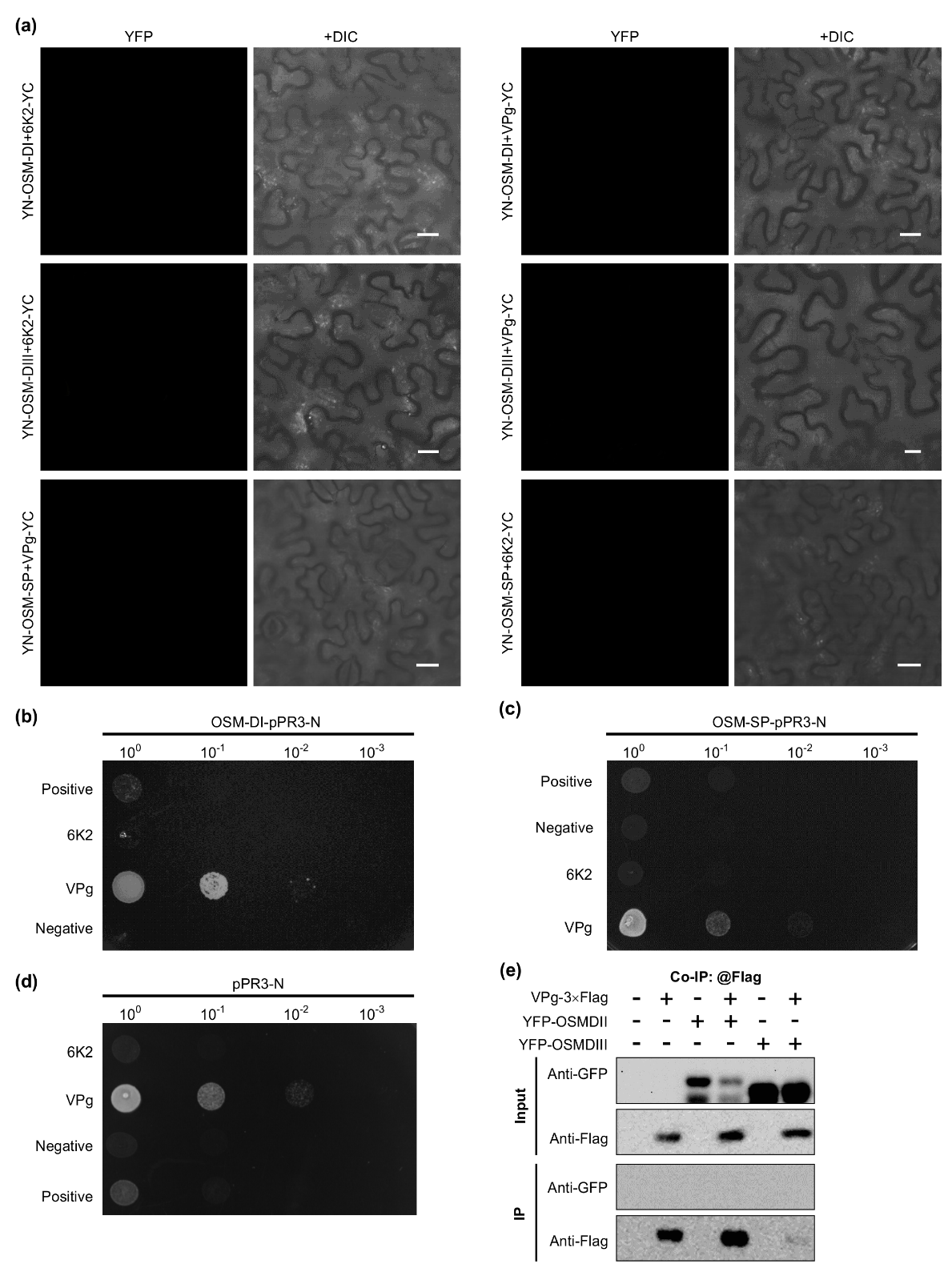
**

**Fig. S10** Transient expression of the AtOSM34 DII deletion mutant on TuMV intercellular movement and viral accumulation. (a) Confocal microscopy observation of viral intercellular movement *in N. benthamiana* leaf tissues. Agrobacterium cells harboring the plasmid TuMV:: GFP//mCherry-HDEL (OD_600_ =0.001) were coinfiltrated into leaves of *N. benthamiana* with empty vector (EV) (upper panels), AtOSM34-Flag-4×Myc (middle panels), or AtOSM34delDII-Flag-4xMyc (lower panels). Red fluorescence emitted by mCherry indicates the mCherry-HDEL expression cassette product, and green fluorescence by GFP indicates the recombinant TuMV-GFP genome product. Cells emitting both red and green fluorescence represent primarily infected ones whereas those emitting GFP only are secondarily infected cells. Images were taken at 66 hpi. Scale bar, 100 µm. (b) RT-qPCR analysis of TuMV genomic RNA accumulation in *N. benthamiana* plants at 68 hpi. Total RNA was extracted from leaves of *N. benthamiana* coinfiltrated with different plasmid combinations shown in (a). Data are means from three independent experiments with SE *(n* = 3) and are presented as arbitrary units relative to the TuMV+EV sample. *NbActin* transcripts in the same sample were used as an internal control. Statistically significant difference, determined by unpaired two-tailed Student's *t*-test comparing the sample treated with TuMV+AtOSM34 and that with TuMV+AtOSM 34delDII is indicated: *, *P* < 0.05.

**
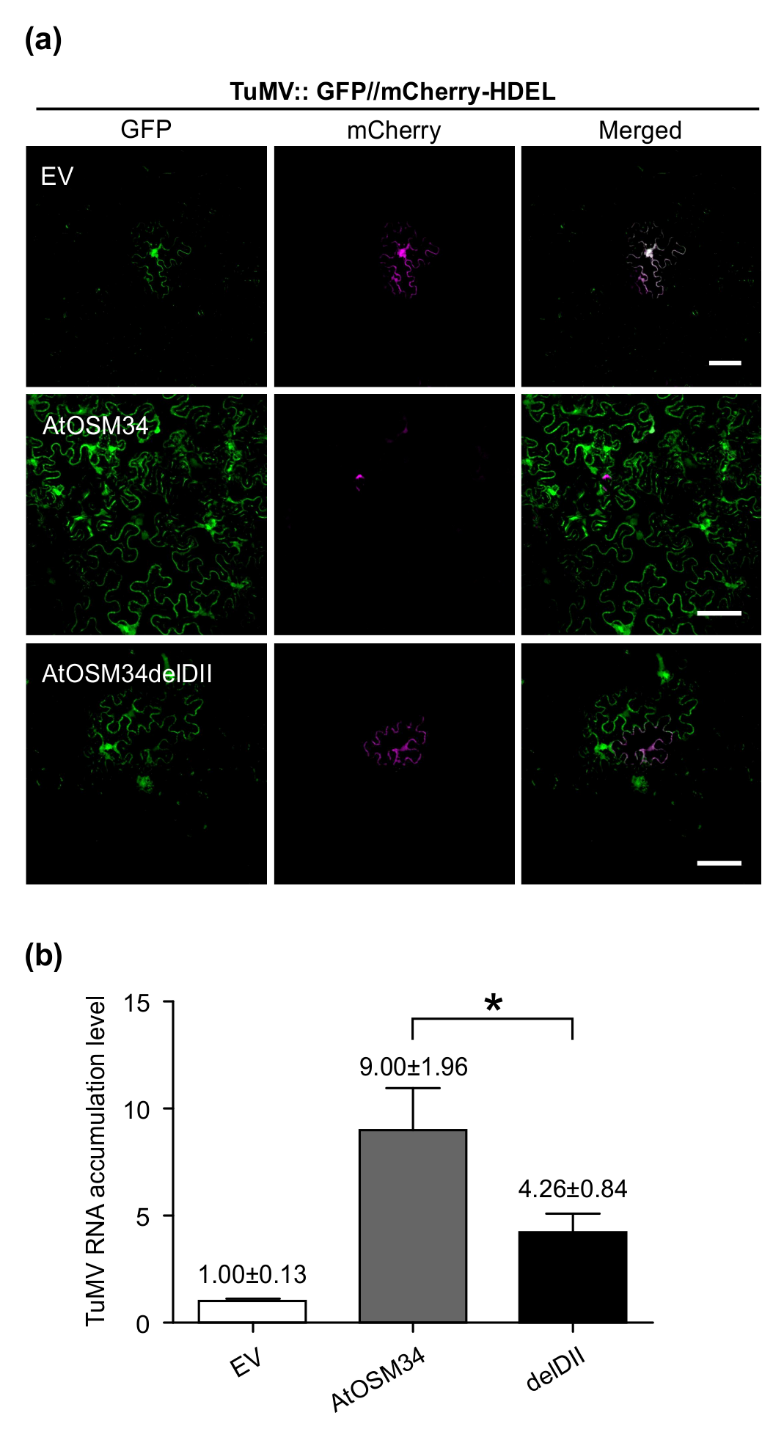
**

**Table S1** List of primers used in this study.

| **Primer names** | **Sequences (5’ to 3’)** | **Notes** |
| --- | --- | --- |
| pPR3-N-AtOSM34-F | GAGTGGCCATTACGGCC atggcaaacctcttggtctct | Primers are designed for cloning into mYTHS vector pPR3-N. The underlined nucleotides indicate *Sfi* I site.  Primers are designed based on the reference sequences (NM_117234.3 for AtOSM34 deposited in GenBank). |
| pPR3-N- AtOSM34-R | GAGAGGCCGAGGCGGCCGttaattctcctcggtgaccatcttg |  |
| BP-AtOSM34-F | GGGGACAAGTTTGTACAAAAAAGCAGGCTTCatggcaaacctcttggtctct | Primers are designed for cloning into the entry vector pDONR221.  GenBank Accession no.: NM_117234.3 |
| BP-AtOSM34-R | GGGGACCACTTTGTACAAGAAAGCTGGGTCattctcctcggtgaccatcttg |  |
| BP-AtOLP-F | GGGGACAAGTTTGTACAAAAAAGCAGGCTTCatgcatatcgaaaaatactt | Primers are designed for cloning into the entry vector pDONR221.  GenBank Accession no.: NM_001336188 |
| BP-AtOLP-R | GGGGACCACTTTGTACAAGAAAGCTGGGTCgtggcagaagatgactttgag |  |
| BP-NbOLP1-F | GGGGACAAGTTTGTACAAAAAAGCAGGCTTCATGGCTTCTTCATCCACAAGA | Primers are designed for cloning into the entry vector pDONR221.  GenBank Accession no.: NbS00012471g0003.1(NbOLP1), NbS00007534g0005.1(NbOLP2) |
| BP-NbOLP2-F | GGGGACAAGTTTGTACAAAAAAGCAGGCTTCATGGCTTCTTCTTCTTCTACA |  |
| BP-NbOLP-R | GGGGACCACTTTGTACAAGAAAGCTGGGTCGTGACAGAAAATGACCTTTAG |  |
| TRV-NbOSM-F | CGGAATTCAACTTCCTCAAAAGCTTCCCC | Primers are designed for cloning into vector pTRV2.  GenBank Accession no.: NbS00045440g0005.1 |
| TRV-NbOSM-R | CGGGATCCACCATAGCCTTGACACTCTAG |  |
| BP-AtOSM34-SP-R | GGGGACCACTTTGTACAAGAAAGCTGGGTCggctgttgcggtggagatgag | For generation of AtOSM34 truncated mutants for each domain. |
| BP-AtOSM34-D1-F | GGGGACAAGTTTGTACAAAAAAGCAGGCTTCatggccacattcgaaatcctaaac |  |
| BP-AtOSM34-D2-F | GGGGACAAGTTTGTACAAAAAAGCAGGCTTCatgaccgcagacataaacggacaa |  |
| BP-AtOSM34-D2-R | GGGGACCACTTTGTACAAGAAAGCTGGGTCtctctgcttaaagaatcttgagta |  |
| BP-AtOSM34-D3-F | GGGGACAAGTTTGTACAAAAAAGCAGGCTTCatggactcctcaggtcgtggccga |  |
| BP-AtOSM34-D3-R | GGGGACCACTTTGTACAAGAAAGCTGGGTCcgtgtttggtggctgtcccca |  |
| BP-AtOSM34-74-R | gttcaaagcgtactcagccaaaaagttacaattggtcctacc | For overlapping PCR to generate AtOSM34-D1 . |
| BP-AtOSM34-103-F | ggtaggaccaattgtaactttttggctgagtacgctttgaac |  |
| BP-AtOSM34-143-R | atagctgtaagcgtcagggcaacatagtatccgatggcagtt |  |
| BP-AtOSM34-195-F | aactgccatcggatactatgttgccctgacgcttacagctat |  |
| atosm34-LP (CS916067) | GCTCGTAATCCCACTCAATCTTG | For PCR genotyping. |
| atosm34-RP (CS916067) | GCTACATGATCCCTGACCGTT |  |
| CS819509-LP | TGTGCAATAAACGAGACAACG |  |
| CS819509-RP | AAGCCACCTTTCTCTAGGACG |  |
| CS850431-LP | CTGCAAGCCTCGAATAAAAAC |  |
| CS850431-RP | AAATTCATTGCCTAACCACCC |  |
| LT6 | AATAGCCTTTACTTGAGTTGGCGTAAAAG |  |
| qPCR-TuMV cp-F | TGGCTGATTACGAACTGACG | Primers are designed based on the submitted NCBI sequence for the TuMV isolate (EF028235.1). |
| qPCR-TuMV cp-R | CTGCCTAAATGTGGGTTTGG |  |
| qPCR-AtActin II-F | CACCACAACAGCAGAGCGGGA | Primers are designed based on the reference sequences (NM_112764.4 for AtACT II deposited in GenBank). |
| qPCR-AtActin II-R | TCCCACAAACGAGGGCTGGA |  |
| qPCR-mCherry-F | CACGGGCTTCTTGGCCTTGT | Primers are designed based on the sequence of PCB301TuMV::GFP//HDEL-mCherry. |
| qPCR- mCherry-R | TCCTCCGAGCGGATGTACCC |  |
| qPCR-AtOSM34-F | CTCCAATGTACTGGCTGGGG | Primers are designed based on the reference sequences (NM_117234.3 for AtOSM34 deposited in GenBank). |
| qPCR-AtOSM34-R | TGTCCGTTTATGTCTGCGGT |  |
| qPCR-AtOLP-F | CCGTGACTCCTCACGAAGGC | Primers are designed based on the reference sequences (NM_001336188 for AtOLP deposited in GenBank). |
| qPCR-AtOLP-R | CAAGCCACCACGTGTCCACT |  |
| qPCR-NbOSM-F | GCAGTGGCCGAGGTAATTGT | Primers are designed based on the reference sequences (GenBank Accession no.: NbS00045440g0005.1). |
| qPCR-NbOSM-R | ATCTAAGGTTACGGCACCCG |  |
| qPCR-AtGST-F | TAATAAAAGTGGCGATGACC | Primers are designed based on the reference sequences (NM_100174.3 for AT1G02930 (AtGSTF6) deposited in GenBank). |
| qPCR-AtGST-R | ACATTCAAATCAAACACTCG |  |
| qPCR-AtCAT-F | AAGTATCCAACTCCGCCTGCTG | Primers are designed based on the reference sequences (NM_119675.4 for AT4G35090 deposited in GenBank). |
| qPCR-AtCAT-R | TGGATGAATCGTTCTTGCCTCTC |  |
| qPCR-AtAPX-F | GCCCTGACATTCCTTTCC | Primers are designed based on the reference sequences (NM_202057.2 for At1g07890 deposited in GenBank). |
| qPCR-AtAPX-R | ACAGCAGCGTATTTCTCG |  |
